# Detecting Photosymbiosis in Fossil Scleractinian Corals

**DOI:** 10.1038/s41598-017-09008-4

**Published:** 2017-08-25

**Authors:** Chiara Tornabene, Rowan C. Martindale, Xingchen T. Wang, Morgan F. Schaller

**Affiliations:** 1 Department of Geological Sciences, Jackson School of Geosciences, University of Texas at Austin, Austin, TX 78712 USA; 2 Department of Geosciences, Princeton University, Princeton, NJ 08544 USA; 3 Department of Earth and Environmental Sciences, Rennselaer Polytechnic Institute, Troy, NY 12180 USA

## Abstract

The evolutionary success of reef-building corals is often attributed to photosymbiosis, a mutualistic relationship scleractinian corals developed with zooxanthellae; however, because zooxanthellae are not fossilized, it is difficult (and contentious) to determine whether ancient corals harbored symbionts. In this study, we analyze the δ^15^N of skeletal organic matrix in a suite of modern and fossil scleractinian corals (zooxanthellate- and azooxanthellate-like) with varying levels of diagenetic alteration. Significantly, we report the first analyses that distinguish shallow-water zooxanthellate and deep-water azooxanthellate fossil corals. Early Miocene (18–20 Ma) corals exhibit the same nitrogen isotopic ratio offset identified in modern corals. These results suggest that the coral organic matrix δ^15^N proxy can successfully be used to detect photosymbiosis in the fossil record. This proxy will significantly improve our ability to effectively define the evolutionary relationship between photosymbiosis and reef-building through space and time. For example, Late Triassic corals have symbiotic values, which tie photosymbiosis to major coral reef expansion. Furthermore, the early Miocene corals from Indonesia have low δ^15^N values relative to modern corals, implying that the west Pacific was a nutrient-depleted environment and that oligotrophy may have facilitated the diversification of the reef builders in the Coral Triangle.

## Introduction

Through photosymbiosis, dinoflagellates, called zooxanthellae, live within the tissue of many modern scleractinian corals. Zooxanthellae photosynthesize within the coral tissue providing corals with most of their energy, while the coral hosts in turn live in shallow, clear waters where zooxanthellae have optimal exposure to sunlight for photosynthesis^1–3^. Although both zooxanthellate and azooxanthellate scleractinians exist and can build reefs, a symbiotic relationship with zooxanthellae is an obvious advantage. Zooxanthellae provide corals with photosymbiotic byproducts (e.g., oxygen and glucose), which allow the corals to calcify at an expedited rate, making zooxanthellate corals more efficient reef-builders than azooxanthellate corals in shallow, oligotrophic conditions^2, 4, 5^. Photosymbiosis is, therefore, considered a key aspect of modern corals and thought to be the main driver of the Triassic expansion and diversification of shallow-water scleractinian corals^1, 6–10^. Nevertheless, since zooxanthellae live within the soft tissue of corals, they are not directly preserved in the fossil record, making it difficult to determine whether fossil corals had symbionts, when photosymbiosis originated, or how this relationship evolved through time.

Morphological characteristics of coral skeletons (corallite size, growth form, and level of corallite integration^11^) are frequently used to infer photosymbiosis in the fossil record and are often used in Phanerozoic compilations of coral-zooxanthellae symbiosis^8, 11–13^. For example, zooxanthellate corals tend to be colonial and have small, highly integrated corallites whereas azooxanthellate corals tend to have solitary growth forms and larger polyps^11^. Additionally, some coral colony morphologies are influenced by the presence of zooxanthellae and are used in the fossil record to demonstrate dependence on sunlight^14^. A delicate plate-like or platy coral morphology, for example, is common in quiet, deep, poorly-lit waters where corals need to maximize their exposure to sunlight for zooxanthellae photosynthesis^14^. Morphological characteristics, however, are not definitive and can be misleading when trying to identify whether extinct species, rather than coral assemblages, were symbiotic^13, 15^. More recently, macroscopic and microscopic growth bands have been used to infer photosymbiosis in corals^9, 10^. Macroscopic density bands, which are inferred to be annual growth bands, have been used to estimate fossil coral growth rates as a proxy for ancient photosymbiosis because modern zooxanthellate corals can grow faster than their azooxanthellate counterparts^9^. Alternatively, the regularity of microscopic skeletal growth bands in the fibrous aragonitic fibrous bundles of coral skeletons has also been proposed as a signature of photosymbiosis^10, 16, 17^. Although both growth band proxies have promise, they can be significantly altered by diagenesis and so can only be used in the most pristine samples^10, 15, 17, 18^.

Two geochemical proxies for ancient photosymbiosis were proposed to resolve the photosymbiotic assignment of contentious fossil corals: the stable oxygen (δ^18^ O) and carbon (δ^13^C) isotope ratios of fossil coral skeletons^7^ and the stable nitrogen isotope ratio (δ^15^N) of the coral skeleton-bound organic matrix^19^. The δ^18^O and δ ^13^C ratios of fossil coral skeletons are, again, greatly influenced by diagenesis^7, 10, 15, 17, 18^. Conversion of aragonite to calcite is common in carbonate systems and can profoundly impact skeletal δ^18^O^15, 18, 20^ making the proxy applicable only to a few, well-preserved fossil corals. The coral skeleton-bound organic matrix, on the other hand, may be protected from diagenesis by the skeletal structure of the fossil and should be (at least partially) preserved along with the fossil itself^21–25^ making the δ^15^N proxy applicable to a wider range of fossil corals.

Organic matrix δ^15^N values differ significantly in modern zooxanthellate corals (3–13‰^19, 25^; all δ^15^N values here are reported with respect to AIR) relative to modern azooxanthellate corals (9–19‰^10, 19, 26^). Differences in organic matrix δ^15^N between zooxanthellate and azooxanthellate corals were originally attributed to the nutritional lifestyle of the coral polyps and the source of nitrogen to the organic matrix of the coral^19^ but recent studies show that the differences are also controlled by the δ^15^N of the nitrogen sources to the corals at the site of growth and nutrient cycling in deep versus shallow waters^25–27^. For example, corals living below the euphotic zone feed on suspended particulate organic matter that has relatively high δ^15^N due to partial decomposition^25^; as a result, their skeletal-bound organic matrix has higher δ^15^N values than zooxanthellate corals living in oligotrophic settings. In the modern ocean this leads to a ~7‰ δ^15^N offset between zooxanthellate and azooxanthellate corals^10, 25–27^.

Since the first application of the δ^15^N proxy, the only fossil corals tested are well-preserved (aragonitic) Triassic corals from Alakir Çay Valley in Turkey, which produce δ^15^N values ranging from 2 to 7‰^10, 19, 24^. Nevertheless, to date, no fossil coral has displayed a high δ^15^N value (i.e. >9‰), equivalent to modern azooxanthellate corals, which raises a fundamental question about the reliability of the proxy in deep time. Additionally, even though diagenesis should not affect skeleton-bound ratios in pristine corals, no tests have been conducted on the δ^15^N of fossil corals with varying levels of recrystallization.

In this study, a suite of modern zooxanthellate and azooxanthellate corals as well as fossil zooxanthellate- and azooxanthellate-like corals^11^ with varying coral morphologies and levels of diagenetic overprint are analyzed for skeletal-bound organic matrix δ^15^N (Table 1). The key objectives are: (1) to evaluate the applicability of the δ^15^N proxy^19^ for ancient photosymbiosis to fossil corals with different morphologies and from varying localities and ages. Zooxanthellate- and azooxanthellate-like fossil samples are expected to plot in different δ^15^N ranges, as they do for modern corals. (2) Determine the effect of diagenetic alteration on the δ^15^N of fossil corals. Samples are screened for diagenesis through macroscopic observations, thin section petrography, micro-Raman spectroscopy, and scanning electron microscopy (SEM); one recrystallized sample was run in conjunction with aragonitic ones. When organic matter was preserved within the original, aragonitic skeleton of the coral, it was assumed to be primary. (3) Compare the original δ^15^N proxy dialysis/combustion method^19^ to the recently developed persulfate/denitrifier method^25^. When enough mass was available (i.e. 15–50 g), a duplicate sample was also processed using the dialysis/combustion method to test for procedural precision.

**Table 1:** Coral Samples Analyzed in This Study.

Taxonomic Identification	Museum Inventory Number	Age	Photosymbiotic Assignment	Collection Site	Curation Site
*Diploria labyrinthoformis*	NPL 73815	Modern	Z	Turks and Caicos	Non-Vertebrate Paleontogy Laboratory, Austin TX
*Favia fragum*	NPL 73816	Modern	Z	Turks and Caicos	Non-Vertebrate Paleontogy Laboratory, Austin TX
*Desmophyllum dianthus*	SS0118	Modern	AZ	Southern Surveyor Dredge	Natural History Museum London
*Diploria strigosa*	NPL 73818	Holocene	Z-Like	Turks and Caicos	Non-Vertebrate Paleontogy Laboratory, Austin TX
*Oulophyllia* sp.	AZ5949	Late Miocene	Z-Like	Bontang, Indonesia	Natural History Museum London
*Acropora papillare*	AZ6977	Early Miocene	Z-like	Kari Orang, Indonesia	Natural History Museum London
*Caryophyllia sp.*	AZ11364	Early Miocene	AZ-like	Kari Orang, Indonesia	Natural History Museum London
*Antiguastrea lucasiana*	GBA 2016/002/0001	Oligocene	Z-Like	Castelgomberto Shale, Italy	Geologische Bundesanstalt Wien
*Gablonzeria* sp.	NPL 73819	Triassic (Norian)	Z-like	Tilkedeligitepe Formation, Turkey	Non-Vertebrate Paleontogy Laboratory, Austin TX
*Distichomeandra* sp. (1)	NPL 73820	Triassic (Norian)	Z-like	Tilkedeligitepe Formation, Turkey	Non-Vertebrate Paleontogy Laboratory, Austin TX
*Distichomeandra sp*. (2)	NPL 73821	Triassic (Norian)	Z-like	Tilkedeligitepe Formation, Turkey	Non-Vertebrate Paleontogy Laboratory, Austin TX

*Zooxanthellate- and azooxanthellate-like morphologies are inferred based on morphological characters^11^. Z = zooxanthellate coral (modern), AZ = azooxanthellate coral (modern), Z-like = zooxanthellate-like coral (fossil), AZ-like = azooxanthellate-like coral (fossil).

Defining a successful proxy for ancient photosymbiosis is important in invertebrate paleontology, carbonate sedimentology, and coral biology as it will provide critical data regarding the evolutionary link between scleractinian corals and symbionts, as well as, the association of coral photosymbiosis and reef-building.

## Results

### Coral Preservation

**Figure 1:**
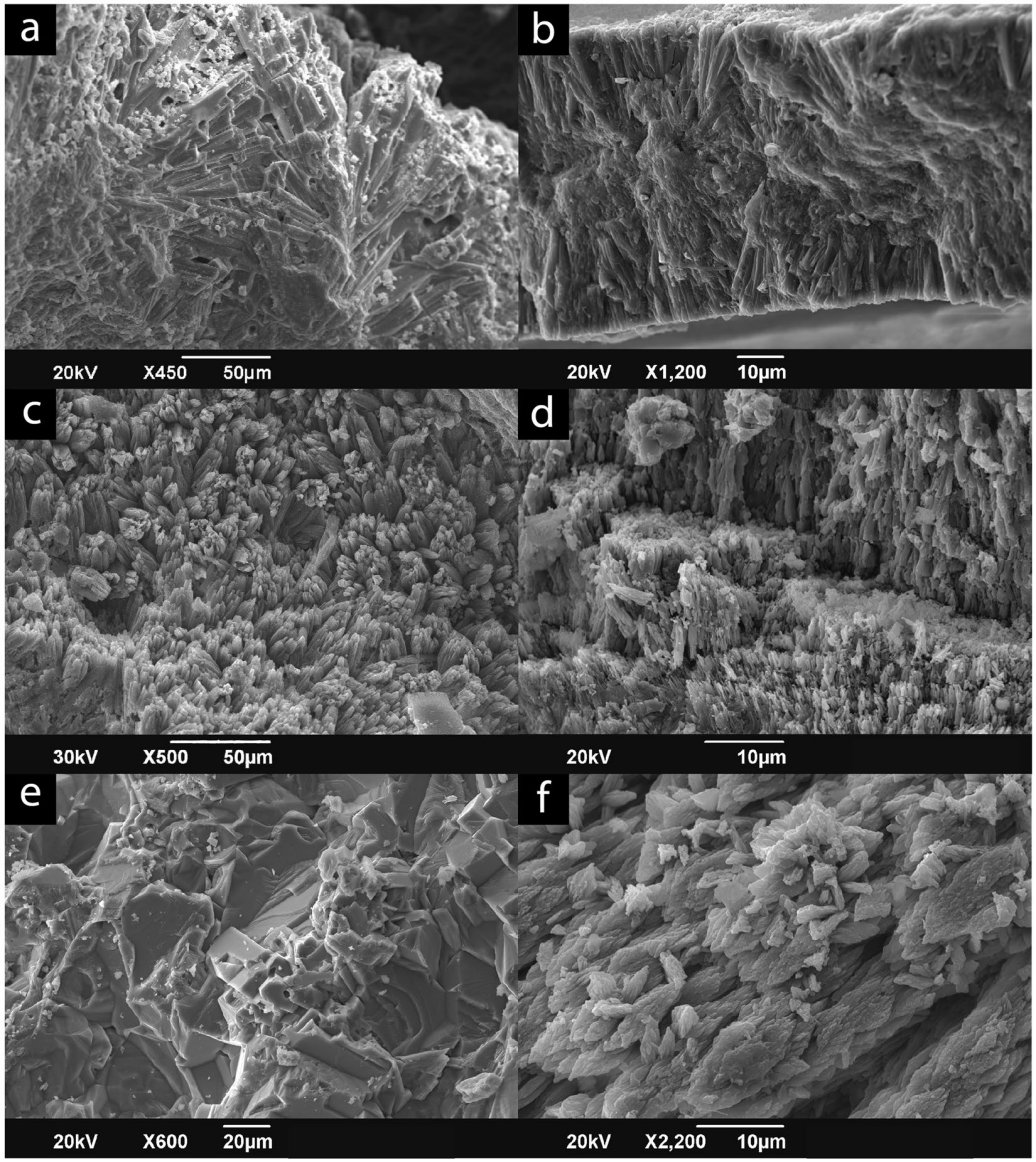
Scanning electron microscope (SEM SE) images of samples; note aragonite bundles. A-B are modern, C is Holocene, D is Miocene, E is Oligocene, and F is Triassic in age. (**a**) Zooxanthellate *Diploria labynthoformis*; (**b**) Azooxanthellate *Desmophyllium dianthus*; (**c**) Zooxanthellate *Diploria strigosa*; (**d**) Azooxanthellate-like *Caryophyllia* sp.; (**e**) Zooxanthellate-like *Antiguastrea lucasiana*; note complete recrystallization to blocky calcite; (**f**) *Distichomeandra* sp. (2).

All of the modern (zooxanthellate and azooxanthellate), Holocene, and Miocene samples, as well as one Triassic sample (*Distichomeandra* sp., sample 2 [herein noted as *Distichomeandra* sp. (2)]) are composed of aragonite. These corals display primary aragonite bundles in scanning electron microscopy (SEM) secondary electron (SE) images, aragonite fans in thin sections, and Raman spectra consistent with aragonite^28^ (Fig. S15), thus we conclude that they have retained their original aragonitic skeletal structure (Fig. 1). Triassic samples *Gablonzeria* sp. and *Distichomeandra* sp. sample 1 [herein noted as *Distichomeandra* sp. (1)] (Table 1) retained their coralline structures, as visible in thin section, but displayed varying levels of diagenesis; both blocky calcite and aragonite bundles were visible on specimens in SEM SE images and thin sections. These samples were, therefore, subjected to a more detailed sample preparation to remove the recrystallized regions prior to analyses (see Methods). The Oligocene coral (*Antiguastrea lucasiana*; Table 1) is completely recrystallized to calcite and is used to evaluate the impact of diagenesis on coral δ^15^N (Fig. 1e). For detailed diagenetic screening summaries see supplemental material.

In general, the thin section petrography and Raman spectroscopy of modern, Miocene, and Triassic corals showed that organic material is disseminated throughout the aragonitic coral skeleton or concentrated in the centers of calcification with no obvious contamination. Only one specimen (*Oulophyllia* sp. AZ5949) had some organic-rich microborings that contained organics (see Fig. S15); so far as it was possible, all borings were removed from coral specimens during sample preparation.

### Organic Matrix Nitrogen Isotope Ratios

**Figure 2:**
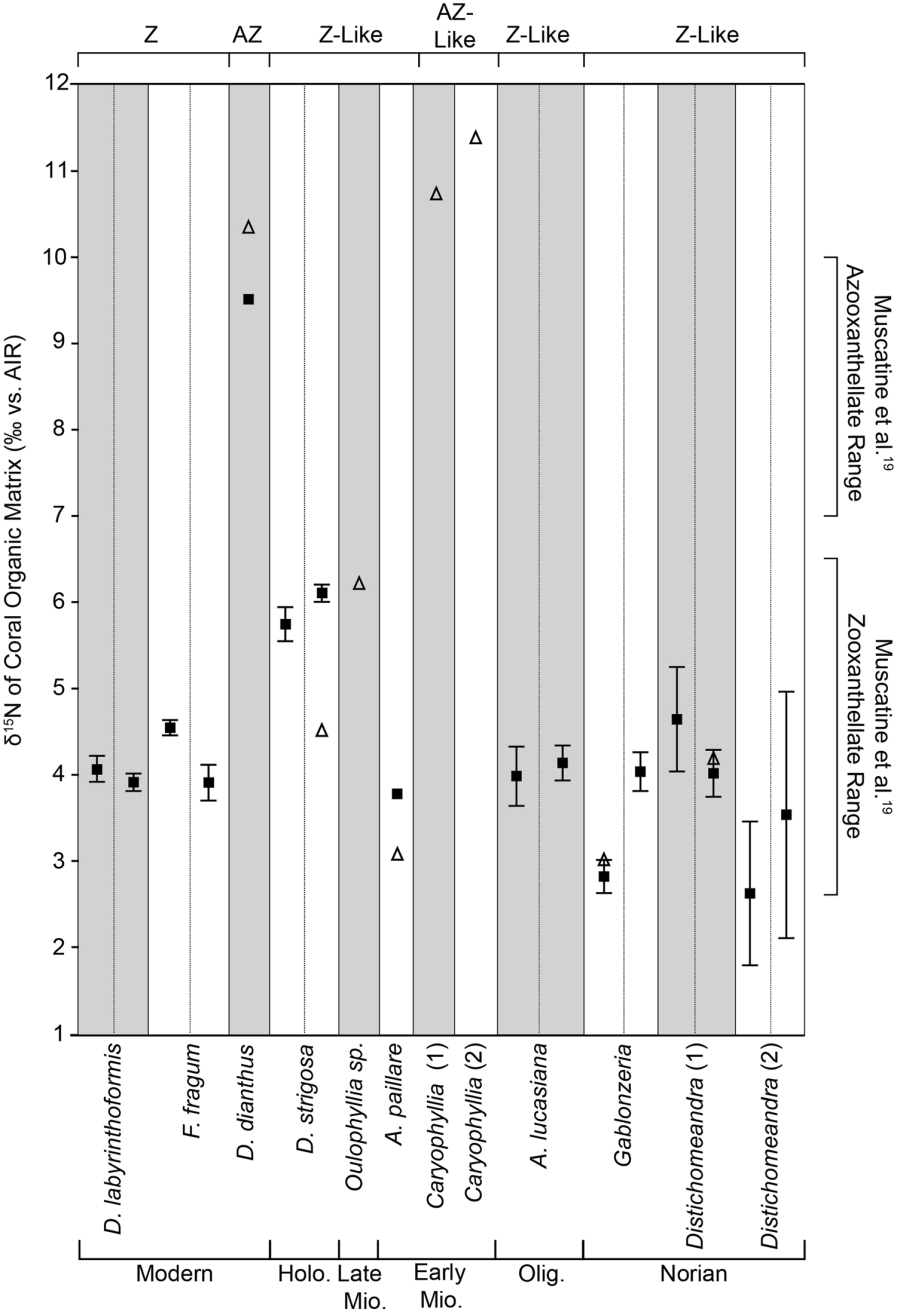
Mean δ^15^N of the organic matrix of coral samples. White and grey boxes differentiate samples, whereas dashed lines separate trials. Black squares mark specimen analyzed using the dialysis/combustion method^19^; triangles mark samples analyzed using the persulfate/denitrifier method^24^. Error bars are one standard deviation from the mean. Z = zooxanthellate coral (modern), AZ = azooxanthellate coral (modern), Z-like = zooxanthellate-like coral (fossil), AZ-like = azooxanthellate-like coral (fossil). Holo. = Holocene, Mio. = Miocene, Olig. = Oligocene.

The skeletal organic matrix δ^15^N values range from 1.90‰ to 6.38‰ (AIR) for modern zooxanthellate and fossil zooxanthellate-like corals (Fig. 2). Standard deviations range from ± 0.10‰ to ± 1.43‰ (Table 2); sample *Distichomeandra* (2)-TR2 displays an unusually high standard deviation of ± 1.43‰, but the standard deviations of all fossil samples analyzed using the dialysis/combustion method are lower than the Triassic sample ( ± 2.72‰) analyzed in the original study^19^. The organic matrix recovered from modern azooxanthellate sample *D*. *dianthus* and Miocene samples were too scarce for replicates (Table 2).

**Table 2:** Nitrogen Isotope Results of Coral Skeleton-bound Organic Matrix δ^15^N.

Samples	Age	Photosymbiotic Assignment	Mean δ^15^N Muscatine Method (‰)	Mean δ^15^N persulfate-denitrifier method (‰)
*Diploria labyrinthoformis*-TR1	Modern	Z	4.07 ± 0.15	—
*Diploria labyrinthoformis*-TR2	Modern	Z	3.92 ± 0.10	—
*Favia fragum*-TR1	Modern	Z	4.55 ± 0.09	—
*Favia fragum*-TR2	Modern	Z	3.91 ± 0.21	—
*Desmophyllum dianthus*	Modern	AZ	9.49	10.30
*Diploria strigosa*-TR1	Holocene	Z-like	5.75 ± 0.20	—
*Diploria strigosa*-TR2	Holocene	Z-like	6.10 ± 0.10	4.42
*Oulophyllia* sp.	Late Miocene	Z-like	—	6.19
*Acropora papillare*	Early Miocene	Z-like	3.86	3.06
*Caryophyllia* sp. (1)	Early Miocene	AZ-like	—	11.30
*Caryophyllia* sp. (2)	Early Miocene	AZ-like	—	10.70
*Antiguastrea lucasiana*-TR1	Oligocene	Z-like	3.99 ± 0.34	—
*Antiguastrea lucasiana*-TR2	Oligocene	Z-like	4.14 ± 0.20	—
*Gablonzeria* sp.-TR1	Norian	Z-like	2.83 ± 0.19	3.02
*Gablonzeria* sp.-TR2	Norian	Z-like	4.05 ± 0.23	—
*Disticomeandra* sp. (1)-TR1	Norian	Z-like	4.65 ± 0.60	—
*Disticomeandra* sp. (1)-TR2	Norian	Z-like	4.03 ± 0.27	4.16
*Disticomeandra* sp. (2)-TR1	Norian	Z-like	2.64 ± 0.84	—
*Disticomeandra* sp. (2)-TR2	Norian	Z-like	3.55 ± 1.43	—

*Error is one standard deviation from the mean. Precision of the persulfate/denitrifier method results is 0.2‰. Samples of the same species analyzed separately are labeled “(1)” and “(2).” Replicates of the same sample are labeled “TR1” and “TR2” respectively; *Desmophyllum dianthus* and the Miocene samples did not yield enough mass for replicates. Zooxanthellate- (Z) and Azooxanthellate-like (AZ) morphologies are inferred based on morphological characters^11^. Z = zooxanthellate coral (modern), AZ = azooxanthellate coral (modern), Z-like = zooxanthellate-like coral (fossil), AZ-like = azooxanthellate-like coral (fossil).

**Figure 3:**
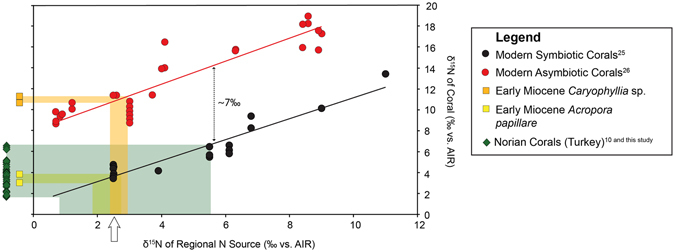
δ^15^N of modern and fossil corals plotted with δ^15^N values of the regional N source. Fossil data are compared to the δ^15^N values of modern corals from different locations, and the δ^15^N of their regional nitrogen source^10, 24, 25^ displaying the typical ~7‰ offset^10^. Early Miocene (18–20Ma) corals (orange and yellow squares) from adjacent sites in Indonesia (note the offset of the Z-like and AZ-like corals) and Triassic corals from Turkey (green rhombi) are plotted outside of the box as the δ^15^N of their regional N source is unknown. The δ^15^N values of Miocene, zooxanthellate coral *Acropora papillare* (yellow squares) and azooxanthellate coral *Caryophyllia* sp. (orange squares) indicate that the δ^15^N of their regional N source was approximately 2.5‰ (arrow), indicative of oligotrophic waters^25^^.^

Several samples were analyzed using two methods: the dialysis/combustion method^18^ and the persulfate/denitrifier method^25^ for comparison (Table 2). Samples analyzed using the newly developed persulfate/denitrifier method have a precision of 0.2‰ and all results except those of *Diploria strigosa*-TR2 fall within one standard deviation of results from the dialysis/combustion method. The modern azooxanthellate coral *Desmophyllum dianthus* δ^15^N value is 9.49‰ using the dialysis/combustion method, and 10.30‰ using the persulfate/denitrifier method (Fig. 2; Table 2) and correlates well with the modern azooxanthellate range (9–18‰^19, 25^). Miocene samples *Oulophyllia* sp. and *Caryophyllia* sp. were too small to be analyzed using the dialysis/combustion method, so they were only analyzed using the persulfate/denitrifier method (Table 2). The δ^15^N values of deep-water azooxanthellate-like *Caryophyllia* sp. samples are similar to those of modern azooxanthellate corals, 11.3‰ and 10.7‰, respectively (Figs 2, 3).

## Discussion

### Coral Organic Matrix δ^15^N as Proxy for Photosymbiosis

A systematic isotopic offset of ~7‰ in coral skeletal organic matrix δ^15^N can be observed between extant zooxanthellate and azooxanthellate corals^10^, ^19^ (Figs 2, 3) The δ^15^N offset can be explained partly as the result of the difference in internal N recycling between symbiotic and asymbiotic corals, and partly by the difference in the δ^15^N of N source to the corals at the site of coral growth^10, 25–27^. For the first time, we report high δ^15^N ratios in two small, solitary deep-water Miocene *Caryophyllia* corals (small solitary forms) similar to those of extant azooxanthellate corals (Figs 2, 3). In contrast, the zooxanthellate-like coral, *Acropora papillare*, from shallower waters recorded much lower values (Figs 2, 3). These data demonstrate that both zooxanthellate and azooxanthellate fossil corals can be distinguished in the fossil record and indicate that the organic matrix δ^15^N has the potential to serve as a successful proxy for photosymbiosis^10, 19^.

Results from Triassic corals from Turkey (1.9–5.2‰; Tables 1, 2) correlate well with previously reported results from the same region^10, 11, 20, 24^. These results corroborate the hypothesis that the δ^15^N of Norian N sources to the Alakir Çay Valley region was low^10^ (Figs 2, 3), indicating that the region was oligotrophic similar to the modern western and subtropical North Atlantic^10, 27^ with a rich diversity of symbiotic corals, including solitary, phaceloid, cerioid, meandroid, and thamnasterioid morphologies^10, 11, 20, 24^.

Recrystallization of fossil corals from aragonite to calcite has been suggested to reduce the amount of organic matrix^21–23^, but not the nitrogen isotope ratio^24^. As a preliminary test, we analyzed a completely recrystallized Oligocene coral, *Antiguastrea lucasiana* (Tables 1, 2; Fig. 1e), which we expected to have a high δ^15^N if diagenesis has a significant impact. This coral, which is zooxanthellate based on its morphology^11^, has δ^15^N values (4.06 ± 0.26‰; Table 2; Fig. 2) consistent with a modern zooxanthellate coral (Fig. 2). This result suggests that the δ^15^N of the coral organic matrix is likely still isolated from the environment during mineralogical phase transition, causing a minimal impact on the δ^15^N values. Preserved insoluble organic matrices have been reported from Pliocene^29^, Cretaceous^30^, and Triassic scleractinian corals^20, 31^ suggesting that the δ^15^N results presented herein most likely represent true organic matrix values and are not diagenetic. Decay of coral organic matrix, however, may lead to lower organic matrix concentrations impacting the precision of the δ^15^N measurements^21, 24, 32–34^. A comprehensive, detailed analysis of the possible biases or possibilities for contamination in ancient organically bound nitrogen archives is beyond the scope of this project, but, overall, the robustness of the δ^15^N proxy indicates that it is more widely applicable as a test for ancient photosymbiosis than the δ^18^O/ δ^13^C isotope proxy of Stanley and Swart^7^.

In order to thoroughly test the impact of diagenesis on this proxy, one would need to use more precise analytical techniques and diagenetic screenings of several specimens with a diagenetic gradient; ideally, one part of their skeleton would be clearly altered (calcitic) and another would be pristine (aragonitic). Thus, if the δ^15^N signatures were identical in these regions, one could conclude that this proxy would be applicable to a wide range of fossil corals, including those that are not perfectly preserved.

### Methodological Comparison

We performed a direct comparison of the original dialysis/combustion method^19^ and the newly developed persulfate/denitrifier method^25^ for analyzing organic matrix δ^15^N in coral skeletons. One significant advantage of the persulfate/denitrifier method is that the sample size requirement is >100 times smaller than the dialysis/combustion method; only 5-10mg of coral carbonate is required for each analysis. This difference in sample size has huge significance for using museum specimens, small corals, or partially altered corals. This method also allows sampling to be more precise so that microborings or other small-scale contaminations can be avoided, which is not always the case with the dialysis/combustion method. Furthermore, in this study the dialysis/combustion resulted in large standard deviations (Table 2) whereas the persulfate/denitrifier method has a precision of 0.2‰^27^.

When enough mass was available (15–50 g) coral samples were processed twice to test the procedural precision of the dialysis/combustion method and were separated in two trials “TR-1” and “TR-2” respectively (Fig. 2). Only the trials of *Favia fragum* and *Gablonzeria* sp. (1) differed by more than 0.5‰ (Table 2). Although the variability between trials may be cause for concern, organic matrix δ^15^N values of modern corals are known to vary over time by up to 3‰^35^. Duplicate samples consist of different parts of the same coral, and so each trial may represent different times of growth; therefore, the difference between the means of the trials is likely an artifact of the large sample size required for the dialysis/combustion method (i.e. 15–50 g^19^).

Results from the persulfate/denitrifier method are within one standard deviation of results from the dialysis/combustion method, except results for *Diploria strigosa*-TR2 (Table 2). This difference may be caused by the fact that the dialysis/combustion method uses dialysis to collect the organic matrix after dissolution of the coral skeleton. Through dialysis, a water-soluble fraction of the organic matrix is lost. In contrast, the persulfate/denitrifier method analyzes the bulk organic matrix including both the soluble and insoluble organic matrix. This fundamental difference in actual analyte could result in differences in the final δ^15^N values^19^. A thorough study of the differences in nitrogen composition of aggregate and soluble organic matrix in fossil corals would be needed to resolve the influence of soluble organic matrix on δ^15^N values. In sum, we suggest that the persulfate/denitrifier method should be the favored technique as it uses less material, can be more precise in the sampling, and results in the analysis of both soluble and insoluble organic matrix.

### Implications for Fossil Coral Paleobiology

The origin of scleractinian photosymbiosis is debated; some ancestral state reconstructions suggest that the first Scleractinia were zooxanthellate^36^, whereas others suggest that scleractinians originated as azooxanthellate^6, 37^. Morphological similarities between modern scleractinians and some tabulate corals (i.e. platy morphology) indicate that algal-coral symbiosis may have existed as early as the Paleozoic^11, 38^, but some authors conclude that Paleozoic corals lacked symbionts^39^. Scleractinians became the dominant reef-builders during the Late Triassic and the similar evolutionary radiation of dinoflagellates and Scleractinia strongly suggest that photosymbiosis evolved during this time^1, 6, 40, 41^. The Triassic fossil corals analyzed herein (Norian age, see Table 1) yielded zooxanthellate-like δ^15^N values (1.9–5.2‰; Figs 2, 3); these results, which are in agreement with the symbiotic assessments of Triassic corals from previous studies^7, 9, 10, 17, 19, 24^ (Fig. 3) corroborate the hypothesis that the evolutionary success of Scleractinia as reef-builders is linked to photosymbiosis, as evidenced by the Late Triassic reef bloom^6, 12, 42^.

Triassic corals could not have been symbiotic with modern zooxanthellae of the genus *Symbiodinium* ^1, 3, 43, 44^. Molecular clock analyses suggest that this genus originated in the early Eocene (~50 Ma), and that the majority of extant lineages diversified during the mid-Miocene^45^ (~15 Ma). If Triassic corals were symbiotic with a different group of dinoflagellates [1, 40], or if Triassic corals did not receive as much nitrogen from their symbionts as modern corals (i.e., if Triassic symbiosis was not as efficient as modern symbiosis), the internal N recycling between the coral host and symbiont would be different from modern corals. Discrepancies in this symbiotic relationship may account for coral skeletal-bound δ^15^N variability, which could cause the distinction between azooxanthellate and zooxanthellate fossil corals to be less clear. For example, N recycling efficiency has been shown to cause up to 2‰ variations in modern corals^27^. A larger dataset of fossil corals from different localities and time periods is needed to refine the confidence intervals as they apply to the fossil record, particularly as there is some overlap in the range of zooxanthellate and azooxanthellae δ^15^N values in modern corals^10, 25, 27^ (Fig. 3).

A better understanding of how zooxanthellate and azooxanthellate corals interacted through their evolutionary history will also provide data about how these corals reacted to environmental perturbations; for instance, a comprehensive assessment of the presence/absence of photosymbiosis in deep time is now possible with the δ^15^N proxy. Ancient analogues of coral reef success, demise, and symbiosis can help ecologists predict how scleractinians will be affected by the current carbon cycle perturbation created by anthropogenic carbon dioxide emissions.

### Nitrogen Cycling in Ancient Oceans

The coral δ^15^N proxy provides not only information on photosymbiosis, but also additional information about the marine nitrogen cycle in the past because coral δ^15^N is affected by regional nitrogen sources to corals^25, 27^ (Fig. 3). In the modern ocean, most zooxanthellate corals live in oligotrophic environments^1, 2, 19^, the cause of which has been attributed to the ecological advantage of coral-zooxanthellae symbiosis with more efficient internal nutrient cycling^1, 2, 10, 19^. A key implication of the Triassic coral results is that the early development and diversification of major fossil reefs happened in oligotrophic oceans^1, 10^.

The modern Coral Triangle is characterized by exceptional ecological diversity^46–48^; however, little is known about its early environment and development^46–48^. We measured the δ^15^N of several well-preserved early Miocene corals from Indonesia collected as part of the THROUGHFLOW Project^48^. Both shallow-water (*Acropora papillare*) and deep-water corals (*Caryophyllia* sp.) from adjacent sites of similar Miocene age (18–20 Ma^48^) were analyzed. The *Acropora papillare* has a δ^15^N of ~3‰ whereas the *Caryophyllia* corals have a δ^15^N of ~11‰ (Table 2; Fig. 3). Despite the small number of samples analyzed (n = 3), this pair of shallow-water and deep-water corals from adjacent sites provides important information about the symbiosis and environment of the Coral Triangle in the early Miocene. First, the *Acropora* sample has a low δ^15^N value, suggesting that this coral was symbiotic^10, 19^ (Figs 2, 3). Second, the δ^15^N values of both *Acropora* and the *Caryophyllia* are among the lowest relative to their modern range respectively (Fig. 3); the calculated δ^15^N of regional nitrate supplied to the euphotic zone was 2.5‰ (Fig. 3), which probably indicate active N fixation in the ambient ocean and a nutrient-depleted environment. Combined, these δ^15^N results indicate that the early Miocene Coral Triangle was a nutrient-depleted environment containing symbiotic corals. It is, thus, possible that the nutrient-depleted environment provided an ecological advantage to symbiotic corals and encouraged rapid diversification within the Coral Triangle.

## Methods

The following corals were analyzed in this study: two modern (<100 years old) zooxanthellate corals, one modern azooxanthellate coral, one zooxanthellate-like Holocene coral, three Miocene corals (two zooxanthellate-like and one azooxanthellate-like), one Oligocene zooxanthellate-like coral, and three Triassic zooxanthellate-like corals (Table 1). The Triassic samples used in this study, *Gablonzeria* sp. and *Distichomeandra* sp., were collected from the same locality as Triassic samples previously studied using the δ^15^N photosymbiosis proxy (Alakir Cay Valley, Turkey)^10, 19, 24^; additional analyses of Triassic fossil corals from Austria can be found in supplemental data. The material analyzed comprises a range of coral morphologies and growth forms; fossil material includes both zooxanthellate and azooxanthellate-like lifestyles inferred for corallite size, growth form, and level of corallite integration^11^ (Table 1).

To assess the diagenetic overprint of the coral samples several techniques were employed including microscopic assessments of polished slabs, thin section petrography, and scanning electron microscopy (SEM), and laser Raman spectroscopy. Specimens were cut perpendicular to corallite growth for thin sections and macroscopic observations. SEM SE images were used to reveal the microstructural composition of the coral skeletons because aragonite consists of needle-like structures, but calcite is blocky in appearance. Samples were coated in gold and photographed in high vacuum in a JEOL JSM-6490LV SEM. Micro-laser Raman spectroscopy was performed using the Bruker 785 nm red laser system at Rensselaer Polytechnic Institute (RPI) on thin sections and grain mounts of carbonate skeletal material and occluded organics in fossil specimens (see supplemental data). Spectra were generated on a 50 um spot, with 5 co-additions of 4 scans each and corrected background; reference spectra on an aragonite standard were performed for comparison. The diagenetic screenings of Triassic samples *Gablonzeria* and *Distichomeandra* (2) revealed infills that were removed prior to analyses (Figs S10, S12).

Sample preparation for nitrogen isotope analyses was conducted following the original dialysis/combustion method^19^ (see Fig. S1). When enough mass was available (i.e. 15–50 g), a duplicate sample was also processed to test for procedural contamination. The original and replicate samples are labeled “TR1” and “TR2” respectively. *Desmophyllium dianthus*, and the Miocene samples did not yield enough mass for replicates.

All specimens were soaked in sodium hypochlorite (commercial bleach, 8.25%) overnight, rinsed with deionized water, and dried at 50 °C overnight. Dried samples were then crushed to a fine powder in a ceramic mortar and pestle. The powder was treated with 2 M Sodium Hydroxide (NaOH) at 80 °C for 15 minutes to remove any residual extrinsic organic material. The samples were rinsed with deionized water through a vacuum filtration system and the cleaned powders were dried in an oven overnight at 50 °C and weighed. The skeletal powders were then decalcified by addition of 4M Hydrochloric acid (HCI). Acid was added until the powder dissolved and the evolution of bubbles ceased. Samples were moisturized with Milli-Q water prior to acid addition to prevent excessive bubble formation. A magnetic mixer was used to stir each solution on a stir plate. The decalcified samples were then neutralized by the addition of 4M NaOH using a glass pipette to achieve a neutral pH of 7. During neutralization, approximate pH values were inferred by applying droplets of each solution onto pH strips. After neutralization, samples were dialyzed using Spectra/Por bags with a molecular-mass cutoff of 3.5 kDa. The Spectra/Por bags were placed in glass containers filled with Milli-Q water that could withhold four times the volume of the sample. The Milli-Q water was changed every 3–4 hours, for a total of four times a day and then left overnight. The dialyzed material was transferred into 24 oz. Nasco sterile Whirl-pak bags. The bags were chosen due to their ability to hold liquid without leakage and their sterility to avoid contamination. The bags were then frozen at −20 **°**C and lyophilized using a Virtis Benchtop SLC freeze dryer.

δ^15^N values were measured using an Elementar Vario Isotope Select Elemental Analyzer connected to a Micromass Isotope Ratio Mass Spectrometer (EA-IRMS) at RPI. In house reference materials as well as IAEA600, SRM1547, and NBS-22 were used as isotope calibration standards. Each freeze-dried sample was divided into triplicates and transferred into tin cups, and were run in blocks bracketed by standards and blanks. Nitrogen isotope ratios were corrected relative to that of the IAEA 600 standard and reported in delta notation in units of parts per thousand or per mil (‰ vs. AIR). Long-term precision on IAEA standards and in-house reference materials on the continuous flow IRMS is 0.2‰ for δ^15^N and 0.03‰ for δ^13^C.

Several samples were analyzed using the newly developed persulfate/denitrifer method at Princeton University. Briefly, the protocol uses 5–10 mg of coral skeleton powder, which is cleaned oxidatively with concentrated sodium hypochlorite solution to remove any external N contamination. After cleaning, the sample is dissolved by addition of 4 M HCl. The resulting organic matter is oxidized into nitrate using a basic potassium persulfate solution and then converted bacterially into nitrous oxide, which can be measured for δ^15^N by gas chromatography-isotope ratio mass spectrometry (for detailed procedure see Wang *et al*.^25^).

### Data Availability

For materials requests and correspondence please contact Chiara Tornabene: ctornabe@utexas.edu or Rowan C. Martindale: martindale@utexas.edu.

## Electronic supplementary material

Supplementary Information
