# Supplementary material for: Detecting Photosymbiosis in Fossil Scleractinian Corals
Source: Sci Rep. 2017 Aug 25;7:9465. doi: 10.1038/s41598-017-09008-4 (PMC5572714; doi:10.1038/s41598-017-09008-4)
Supplement: Supplementary file 1 — Supplementary Information [file 41598_2017_9008_MOESM1_ESM.pdf]

Supplemental Materials For:

Detecting Photosymbiosis in Fossil Scleractinian Corals

Chiara Tornabene, Rowan C. Martindale, Xingchen T. Wang, Morgan F. Schaller

This file includes:

- Fig. S1- Flowchart describing the different procedural steps for the dialysis/combustion method
- Fig. S2-Screening summary of modern zooxanthellate *Diploria labyrinthoformis**
- Fig. S3-Screening summary of modern zooxanthellate *Favia fragum**
- Fig. S4-Screening summary of modern azooxanthellate *Desmophyllum dianthus**
- Fig. S5-Screening summary of Holocene zooxanthellate *Diploria strigosa**
- Fig. S6- Screening summary of Late Miocene zooxanthellate-like coral *Oulophyllia* sp. *
- Fig. S7- Screening summary of Early Miocene zooxanthellate-like coral *Acropora papillare**
- Fig. S8- Screening summary of Early Miocene zooxanthellate-like coral *Caryophyllia* sp. *
- Fig. S9- Screening summary of Oligocene zooxanthellate-like coral *Antiguastrea lucasiana**
- Fig. S10- Screening summary of Norian zooxanthellate-like coral *Gablonzeria* sp. *
- Fig. S11- Screening summary of Norian zooxanthellate-like coral *Distichomeandra* sp. (1)*
- Fig. S12- Screening summary of Oligocene zooxanthellate-like coral *Distichomeandra* sp. (2) *
- Fig. S13- $\delta^{15}N$ of all corals analyzed in study plotted with $\delta^{15}N$ values of the source water.
- Fig. S14- Map of Miocene coral sample collection sites.
- Fig. S15- Selected Raman spectroscopy results with photomicrographs.
- Table S1- Results from nitrogen isotope analyses.

* Note that thin sections of corals (used in petrography, SEM, and Raman spectroscopy analyses) were made before the coral cleaning procedures so some contain material that was removed from the specimen before geochemical analysis.

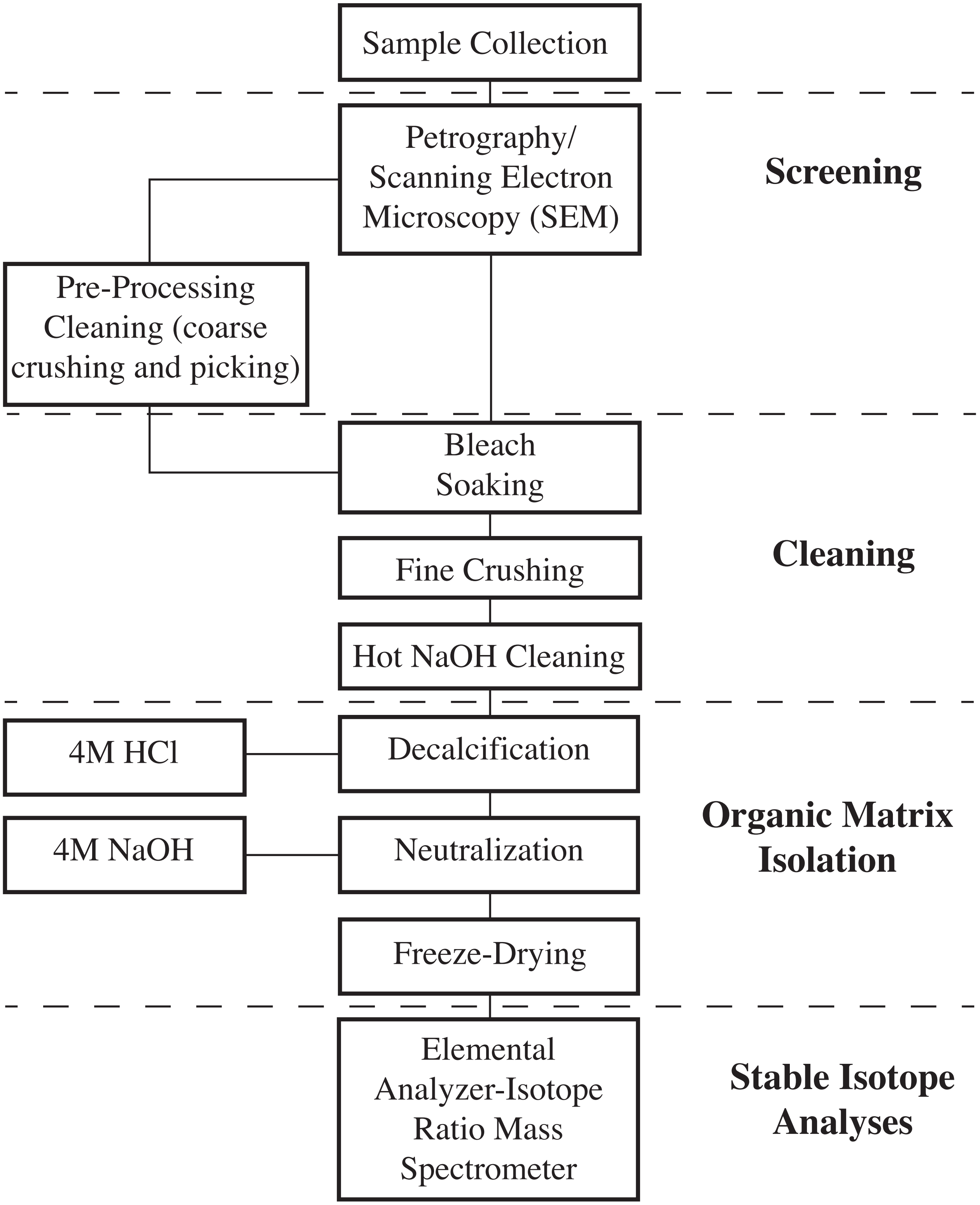

Figure S1- Flowchart describing the procedural steps of the dialysis/combustion method for N analyses.

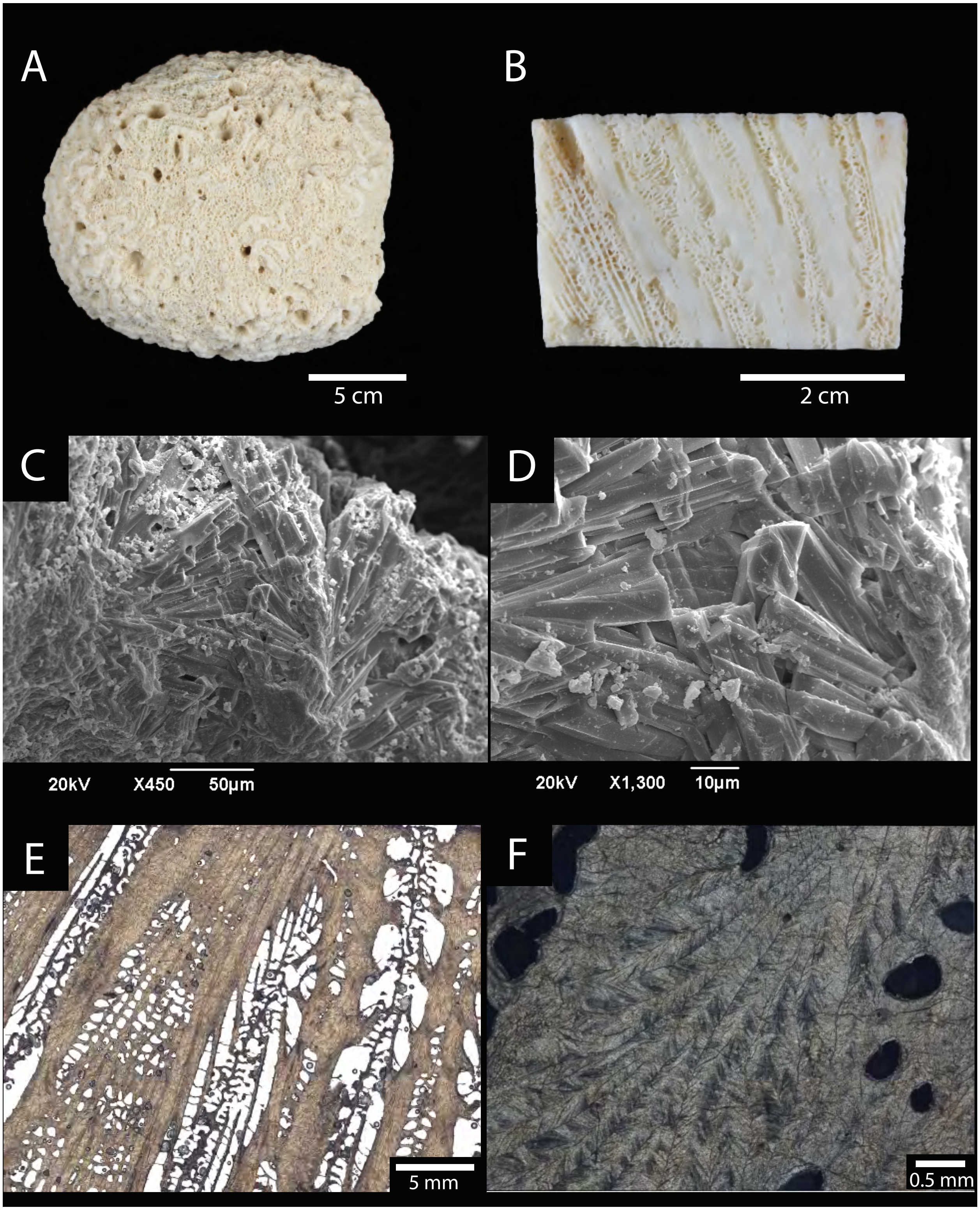

Figure S2- Screening summary of modern zooxanthellate *Diploria labyrinthoformis* (NPL73815). A) Photo of sample prior to analyses. B) Polished cross-section; mirror image of thin section. C-D) SEM SE images, note primary aragonite bundles visible throughout specimen. E-F) Photomicrograph (transmitted light, PPL) of thin section parallel to corallite growth. 5x magnification in PPL(E) and 10x magnification in cross-polarized light (XPL) (F). Coral sample is composed of primary aragonite throughout.

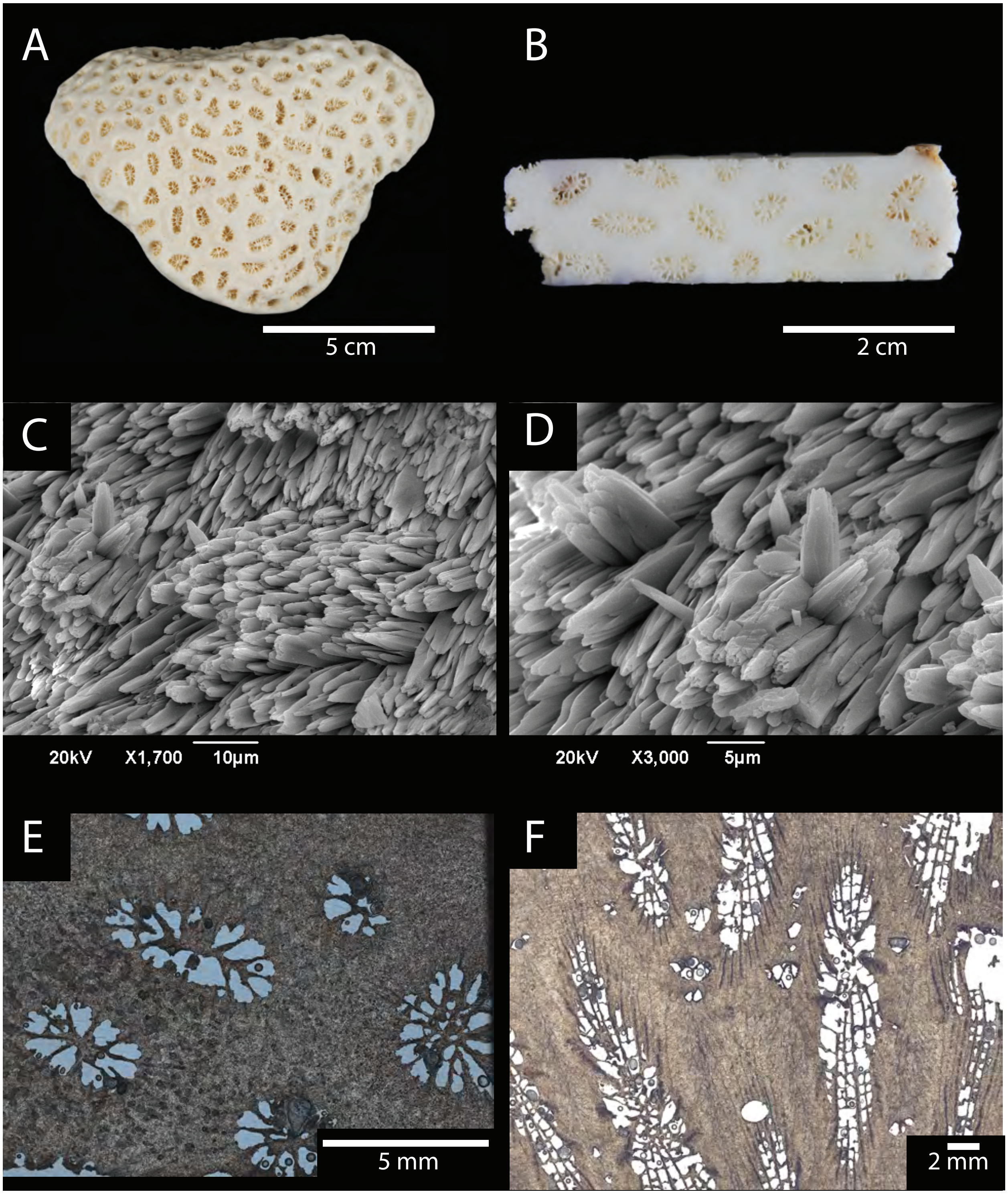

Figure S3- Screening summary of modern zooxanthellate *Favia fragum* (NPL73816). A) Photo of sample prior to analyses. B) Slab cut perpendicular to corallite growth. C-D) SEM SE images, note primary aragonite bundles visible throughout specimen. E) Photomicrograph of thin section perpendicular to corallite growth, 5x magnification in XPL. (F) Photomicrograph of thin section parallel to corallite growth, 5x magnification in PPL. Coral sample is composed of primary aragonite throughout.

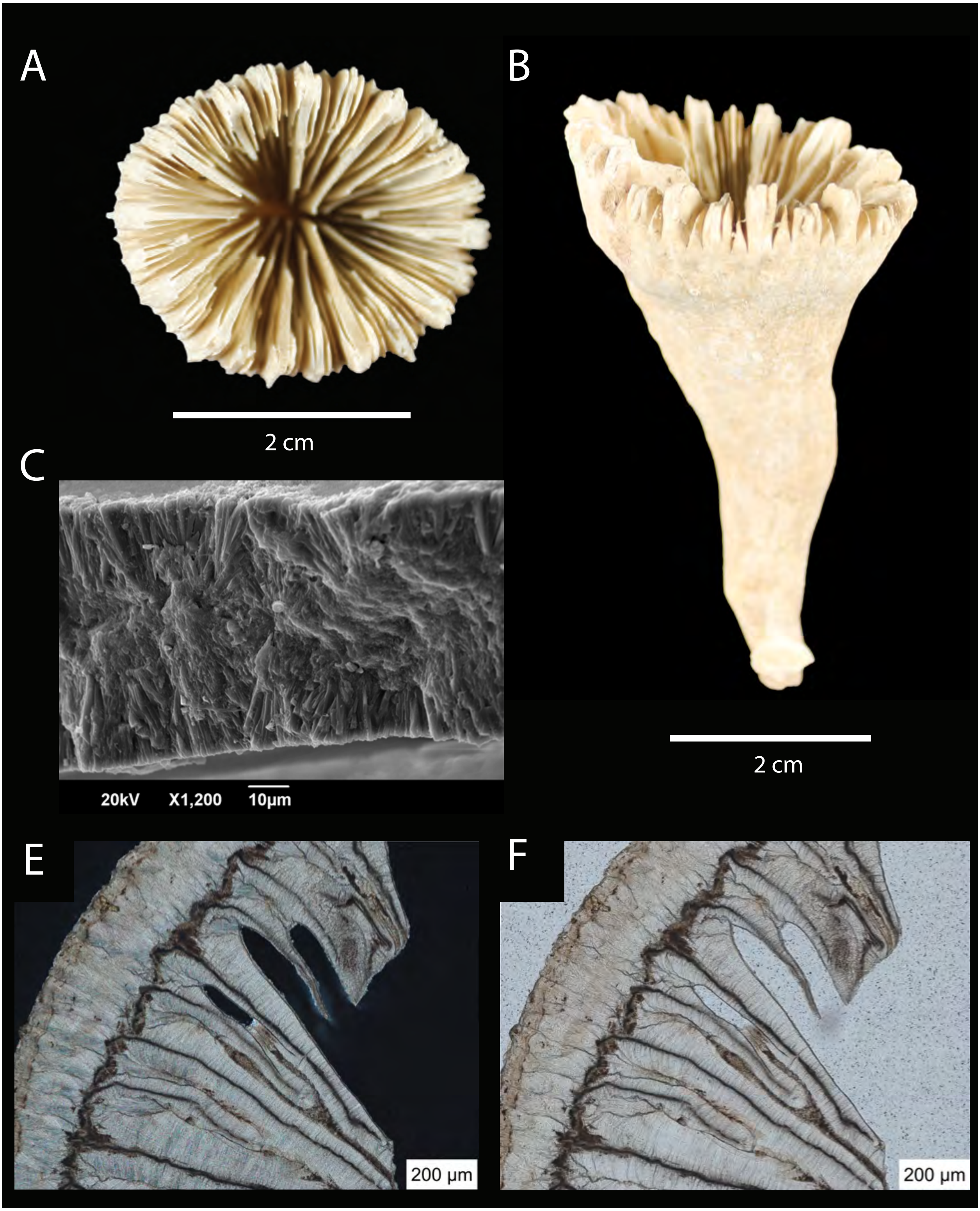

Figure S4- Screening summary of modern azooxanthellate *Desmophyllium dianthus* (SS0118) A-B) Picture of samples prior to analyses. C) SEM SE image characteristic of specimen; primary aragonite bundles visible throughout specimen. D-E) Photomosaic of thin section perpendicular to corallite growth. 10x magnification in XPL (D) and in PPL (E).

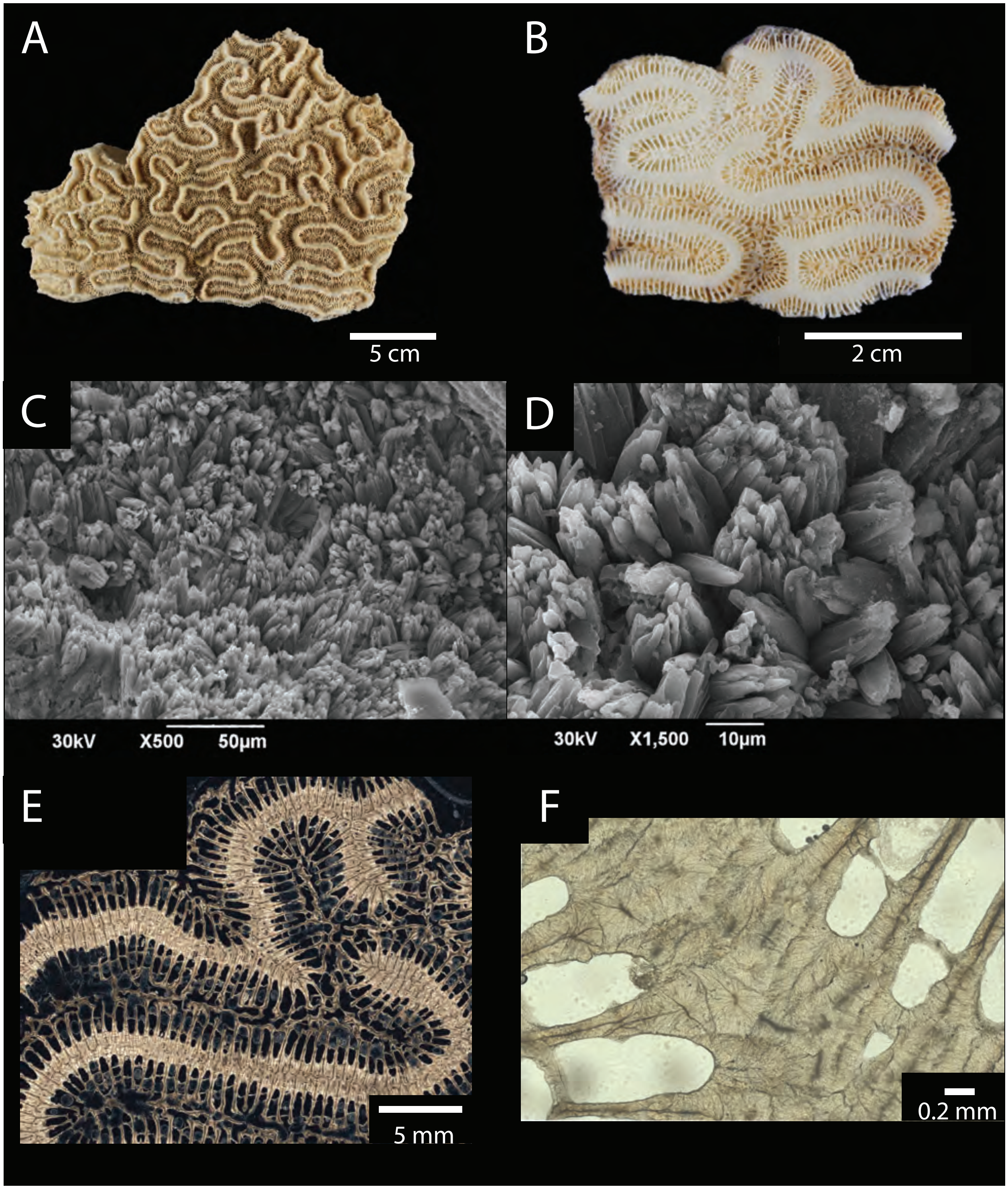

Figure S5- Screening Summary of *Diploria strigosa* (NPL73818) A) Picture of sample prior to analyses. B) Polished cross-section; mirror image of thin section. C-D) SEM SE images of aragonite bundles characteristic of specimen. E) Photomicrograph of thin section perpendicular to corallite growth; 5x magnification (XPL). F) Photomicrograph of thin section perpendicular to corallite growth displaying clear aragonite fans and centers of calcification; 20x magnification (PPL). Coral is composed of primary aragonite throughout.

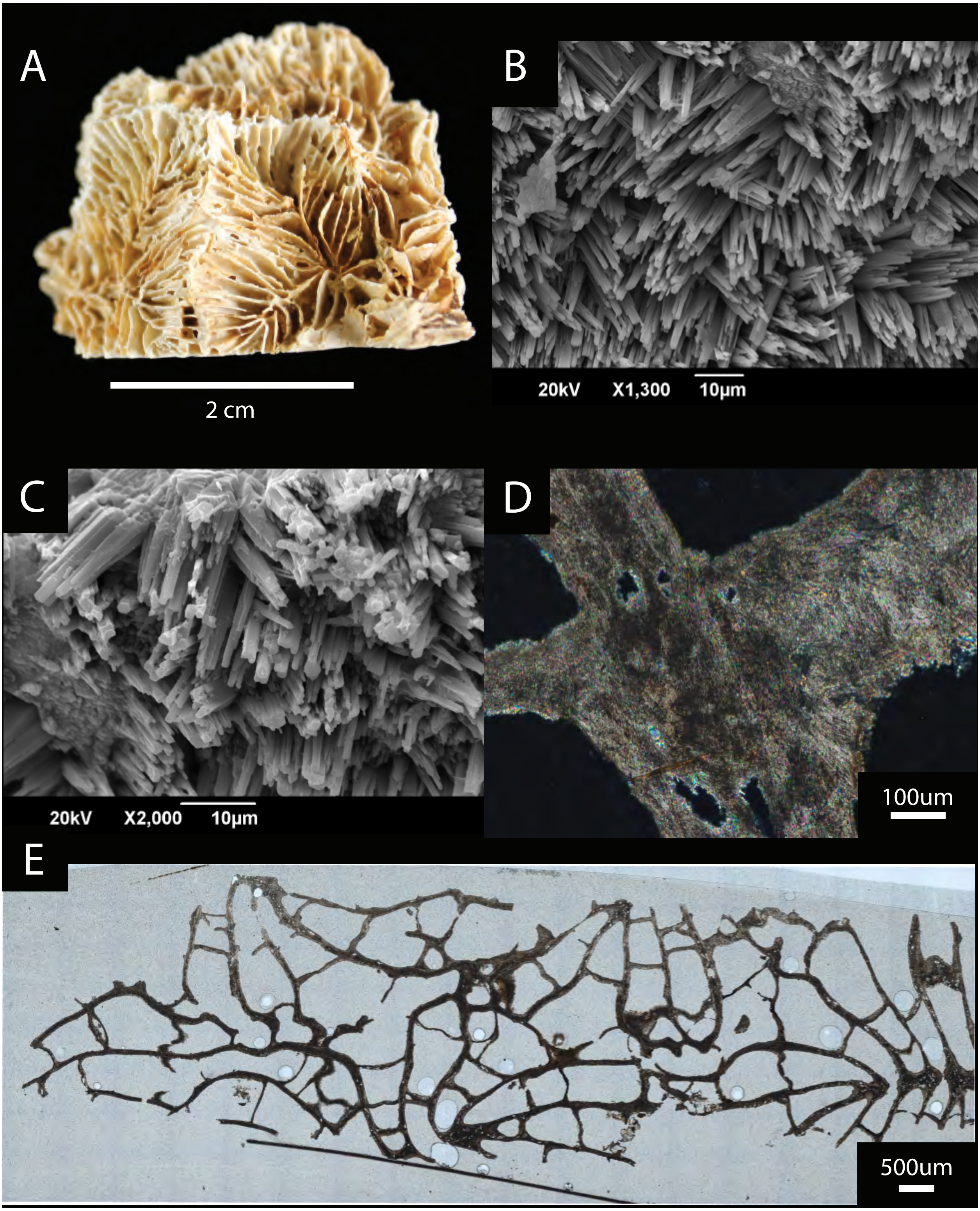

Figure S6- Screening summary of Late Miocene zooxanthellate-like *Oulophyllia sp*. (AZ5949) A) Picture of sample prior to analyses. B-C) SEM SE image characteristic of specimen. D-E) Photo-micrograph of thin section perpendicular to corallite growth. 10x magnification in XPL (D) 5x magnifi-cation in PPL (E).

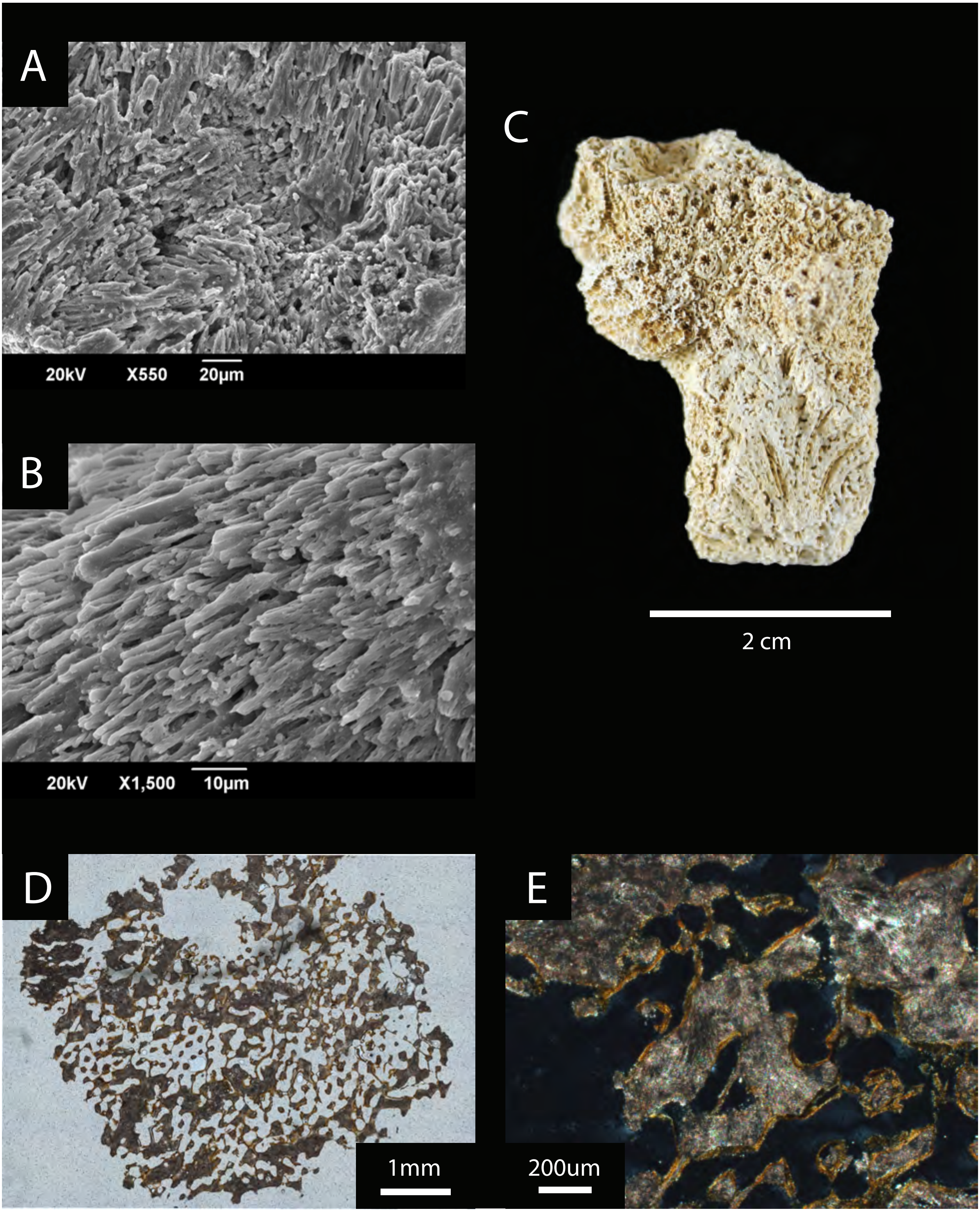

Figure S7- Screening summary of Early Miocene zooxanthellate-like *Acropora papillare* sp. (AZ6977) A-B) SEM SE image characteristic of specimen. C) Photo of specimen prior to analyses. D-E) Photomicrograph of thin section perpendicular to corallite growth. 5x magnification in PPL (D) 10x magnification in XPL (E).

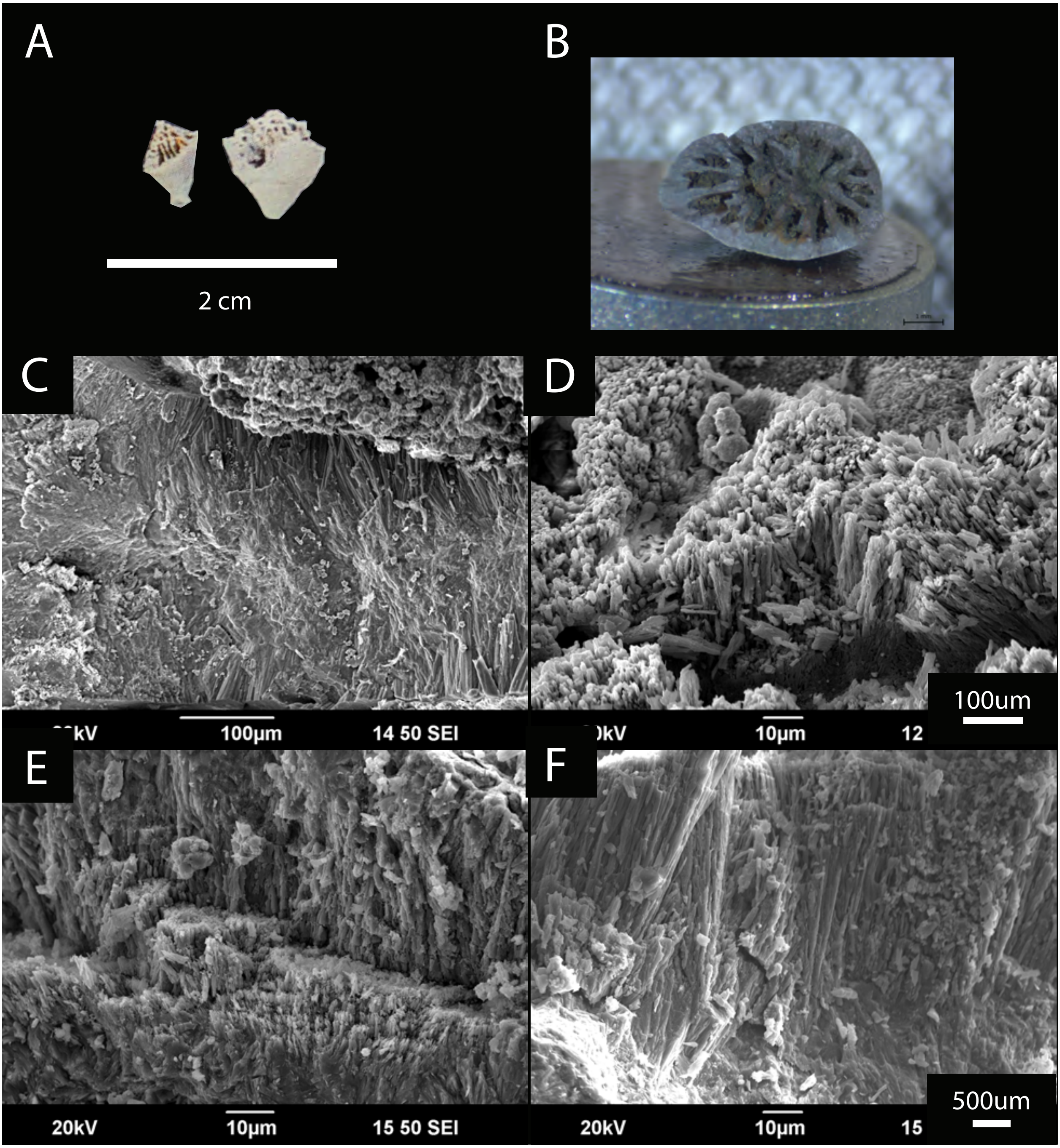

Figure S8- Screening summary of Early Miocene azooxanthellate-like *Caryophyllia* sp. (AZ11364) A) Picture of sample *Caryophyllia* sp. (1) on right and *Caryophyllia* sp. (2) on left prior to analyses. B) Photo of *Caryophyllia* sp. (2) coated in gold and mounted on slide for SEM imaging. C-D) SEM SE images characteristic of *Caryophyllia* sp. (1). E-F) SEM SE images characteristic of *Caryophyllia* sp. (2).

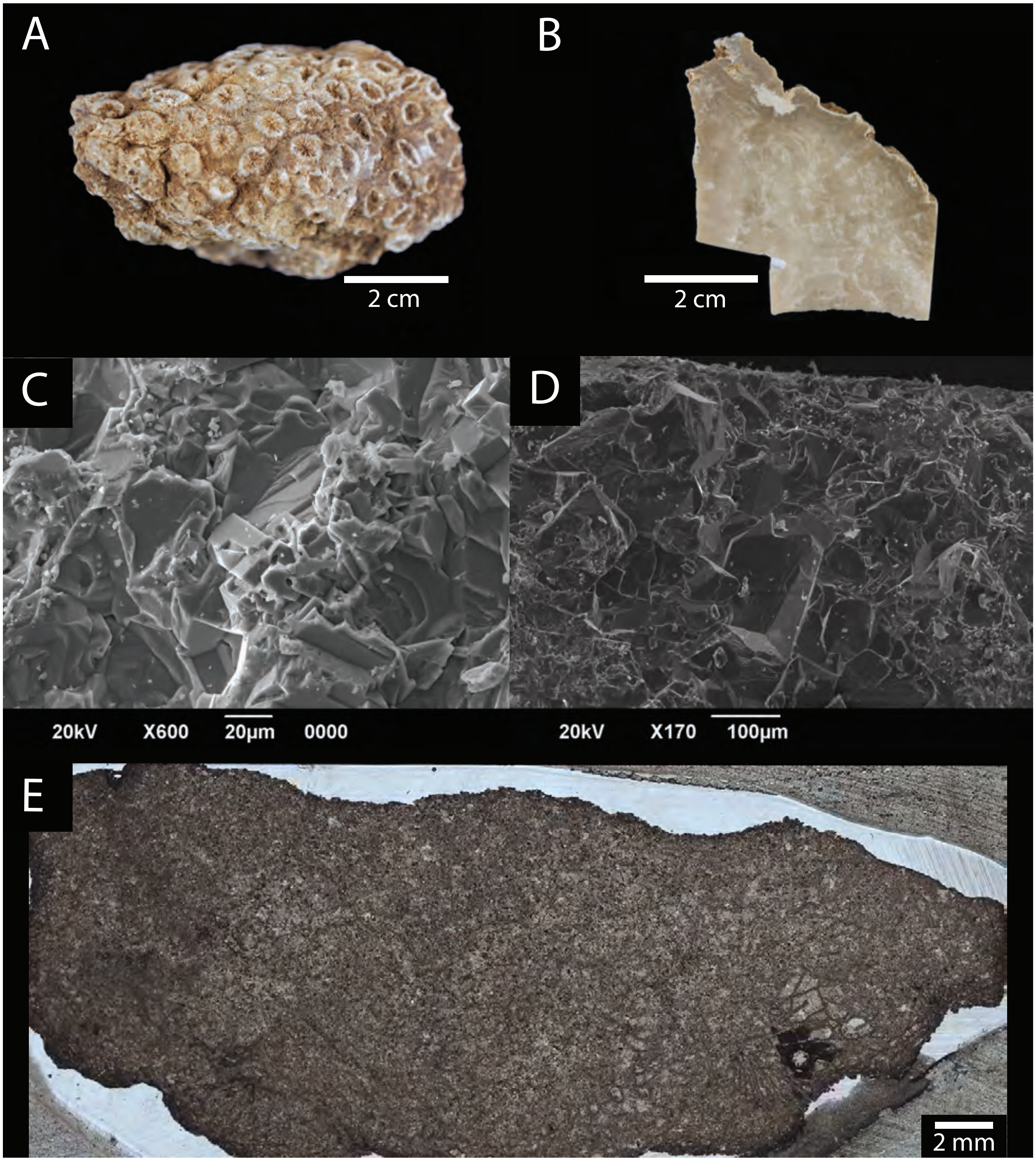

Figure S9- Screening summary of Z-like Oligocene *Antiguastrea alveolaris* (GBA2016/002/0001). A) Picture of sample prior to analyses. B) Polished cross-section. C-D) SEM SE images characteristic of specimen. E) Photomicrograph of thin section perpendicular to corallite growth; 5x magnification in XPL. Diagenesis completely obliterated original coral structure; only blocky calcite structures are visible on the specimen.

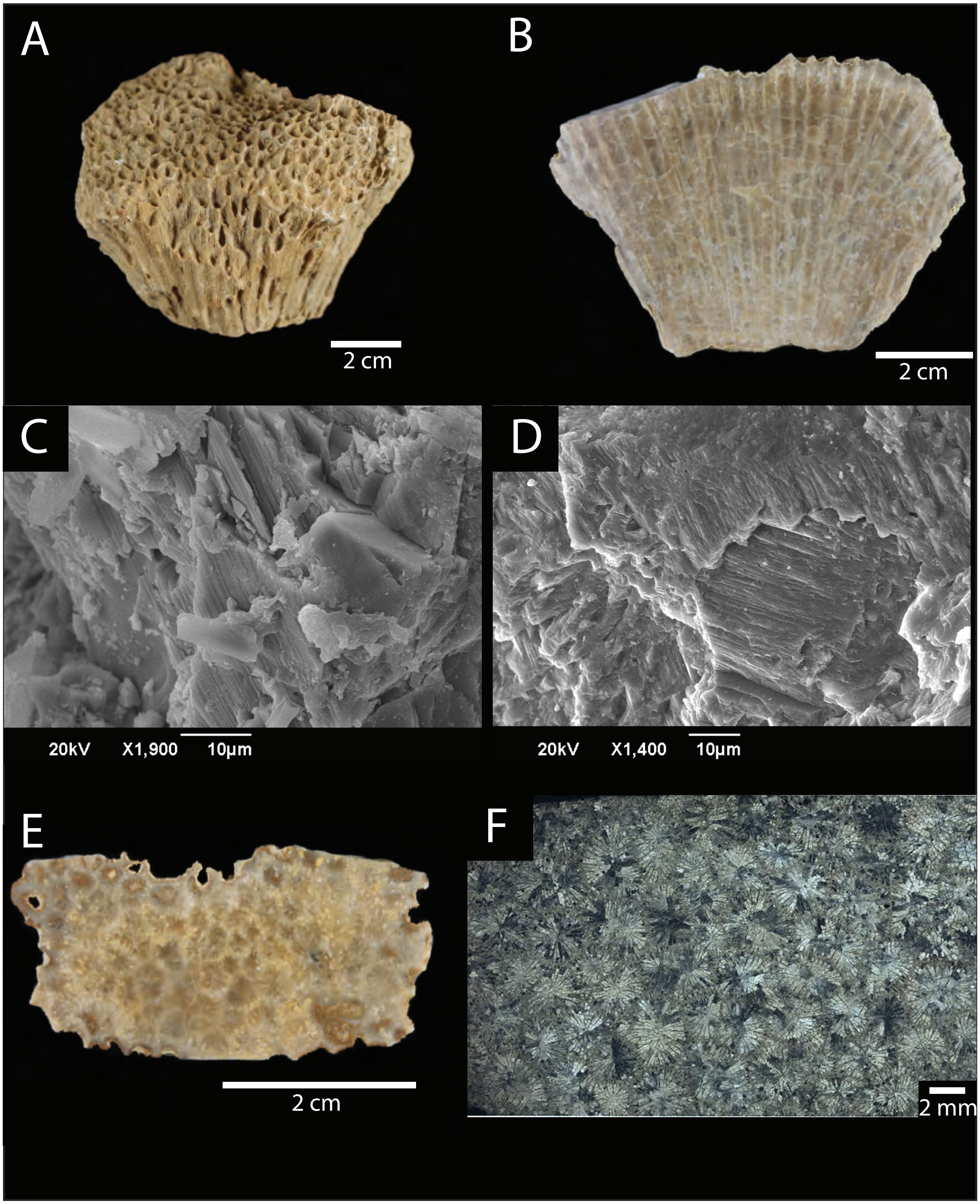

Figure S10- Screening summary of zooxanthellate-like Norian *Gablonzeria* sp. (NPL73819) A) Picture of sample prior to analyses. B) Polished cross-section. C-D) SEM SE images characteristic of specimen. E) Polished slab perpendicular to corallite growth; mirror image of thin section. F) Photomicrograph of thin section perpendicular to corallite growth; 5x magnification in XPL. Primary aragonite bundles are mostly preserved, but are sometimes covered by blocky calcite.

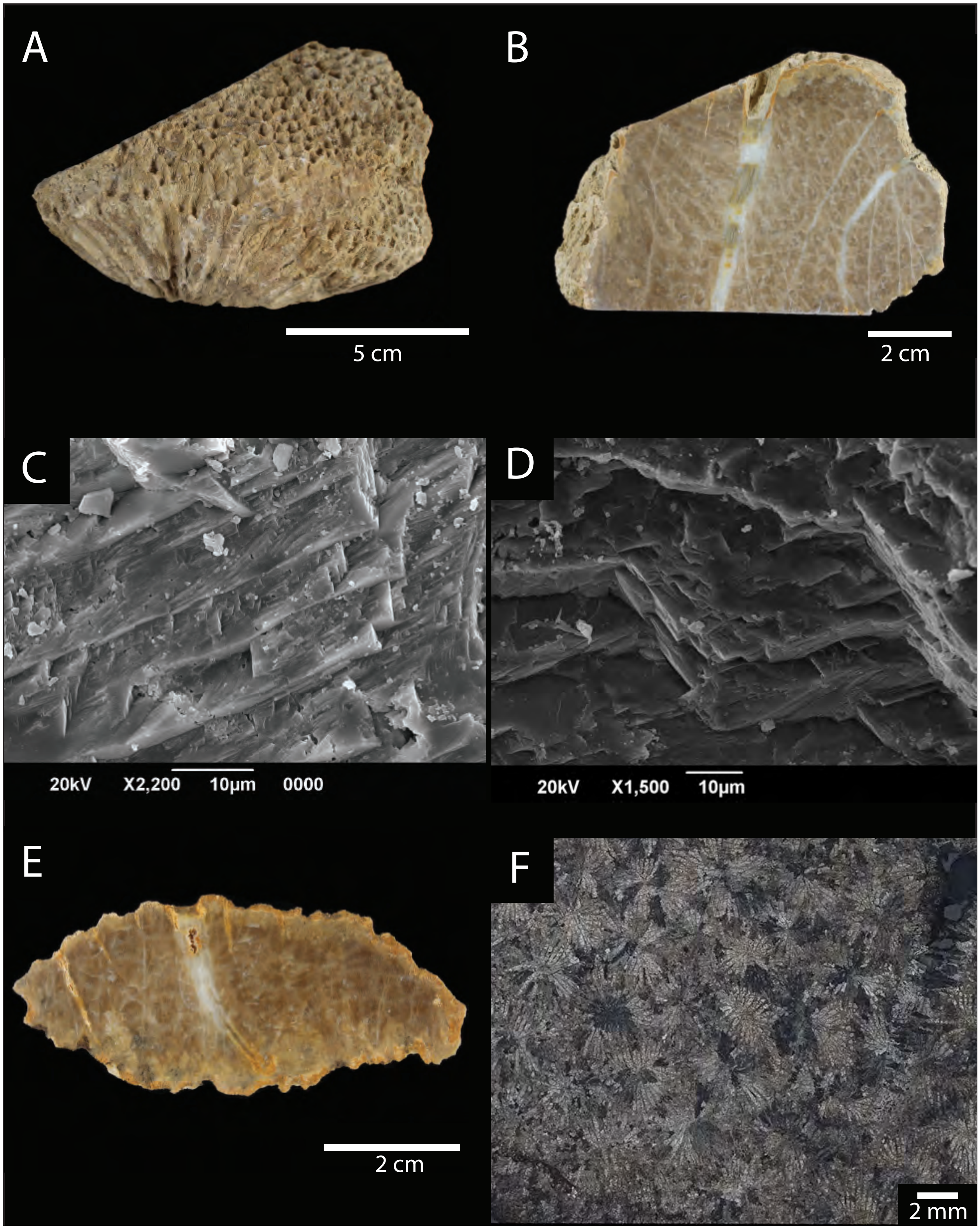

Figure S11- Screening summary of zooxanthellate-like Norian *Distichomeandria* sp. (1) (NPL73820). A) Picture of sample prior to analyses. B) Polished cross-section revealing infill, which was removed prior to analyses. C-D) SEM SE images characteristic of specimen. E) Polished slab perpendicular to corallite growth; mirror image of thin section. F) Photomicrograph of thin section perpendicular to corallite growth. 5x magnification in XPL. Original coralline structure is preserved, but replaced by calcite.

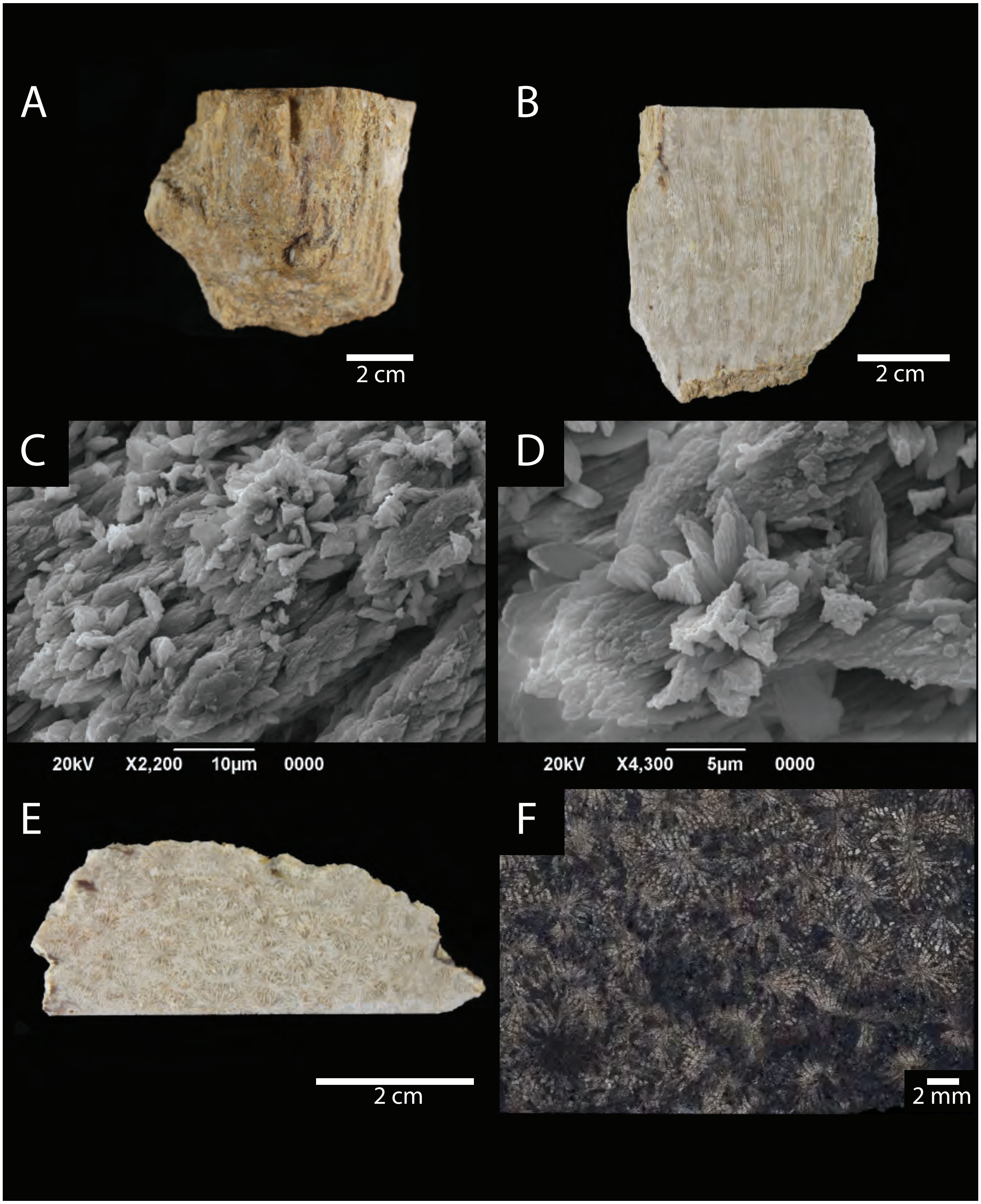

Figure S12- Screening summary of zooxanthellate-like Norian *Distichomeandra* sp. (2) (NPL73821). A) Photo of sample prior to isotope analyses. B) Cross-section parallel to corallite growth. C-D) SEM SE images characteristic of specimen. E) Polished cross-section perpendicular to corallite growth; mirror image of thin section. F) Photomicrograph of thin section perpendicular to corallite growth; 5x magnification in XPL. Original corallite structure is preserved.

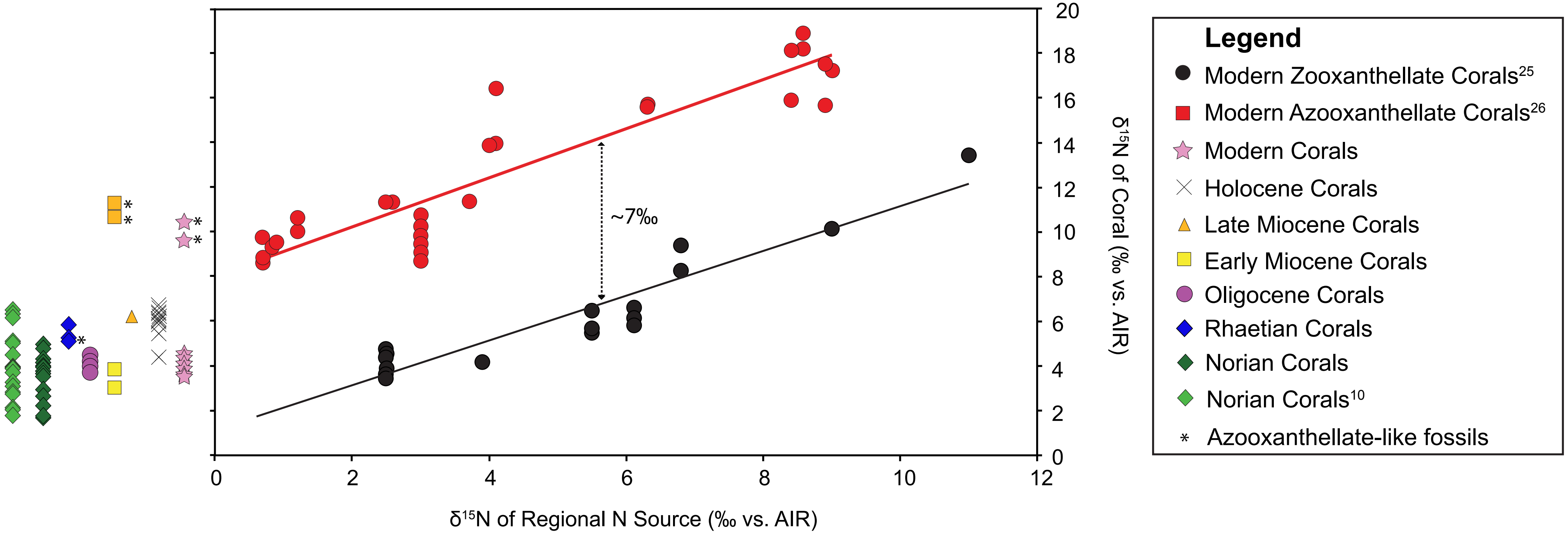

Figure S13- $\delta^{15}N$ of modern and fossil corals plotted with $\delta^{15}N$ values of the regional N source. Data from this study are compared to the $\delta^{15}N$ values of modern corals from different localities, and the $\delta^{15}N$ of their regional N source[10,24,25], which display a ~7‰[10] offset. Results from Norian samples from Turkey are also compared with results from Frankowiak et al.[10] (light green rhombi). Rhaetian samples include *Distichophyllia norica* and *Retiophyllia norica* from Fischerwiese in Austria. They were analyzed as part of this study, but were excluded from publication due to possible contamination (i.e. weathering and recrystallization).

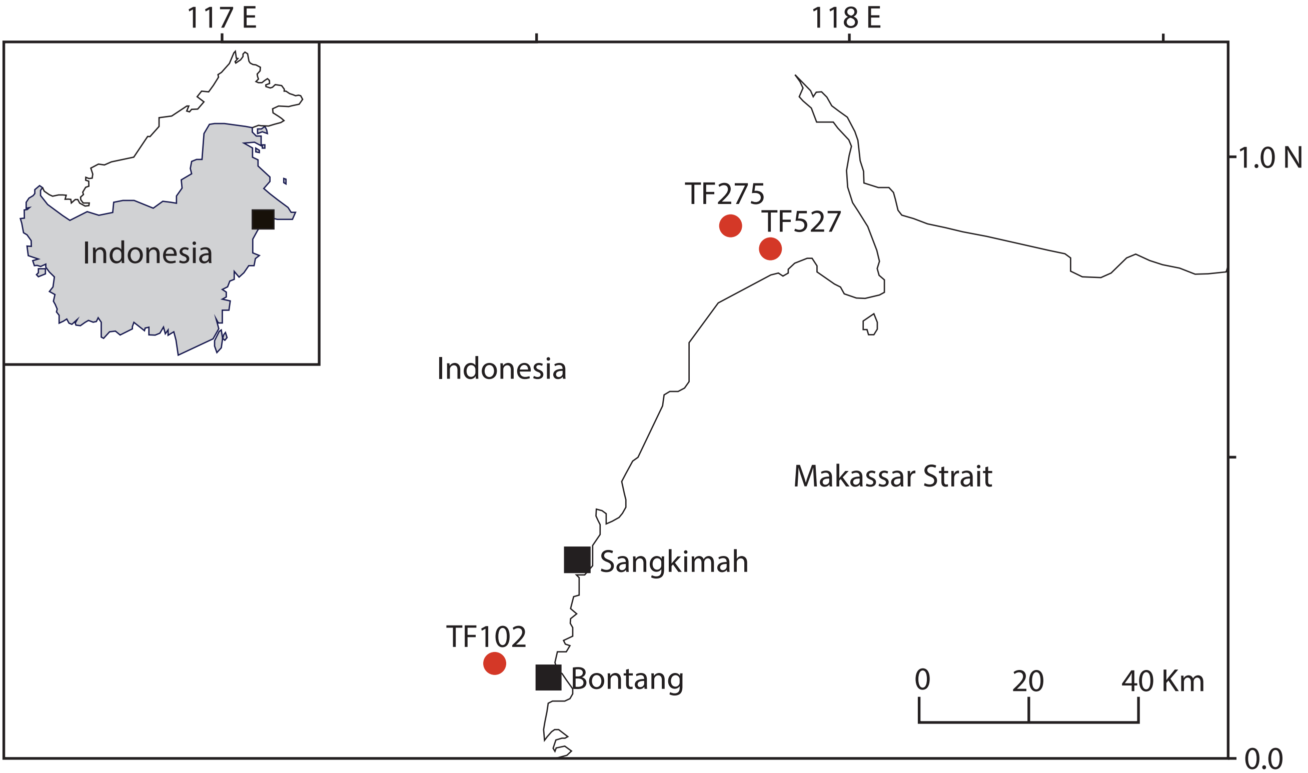

Figure S14- Map of collection sites of Miocene samples (red circles). Late Miocene zooxanthellate-like *Oulophyllia* sp. was collected at locality TF102. Early Miocene zooxanthellate-like *Acropora papillare* was collected at locality TF275, while azooxanthellate-like *Caryophyllia* sp. was collected at locality TF527. Locality data was provided by the Natural History Museum of London; for detailed collection infomation consult Renema et al.[50]. Map data: ©2017 Google. Traced using Adobe Illustrator CC (2017). Retrieved from https://goo.gl/maps/ByUfqfuPPE62.

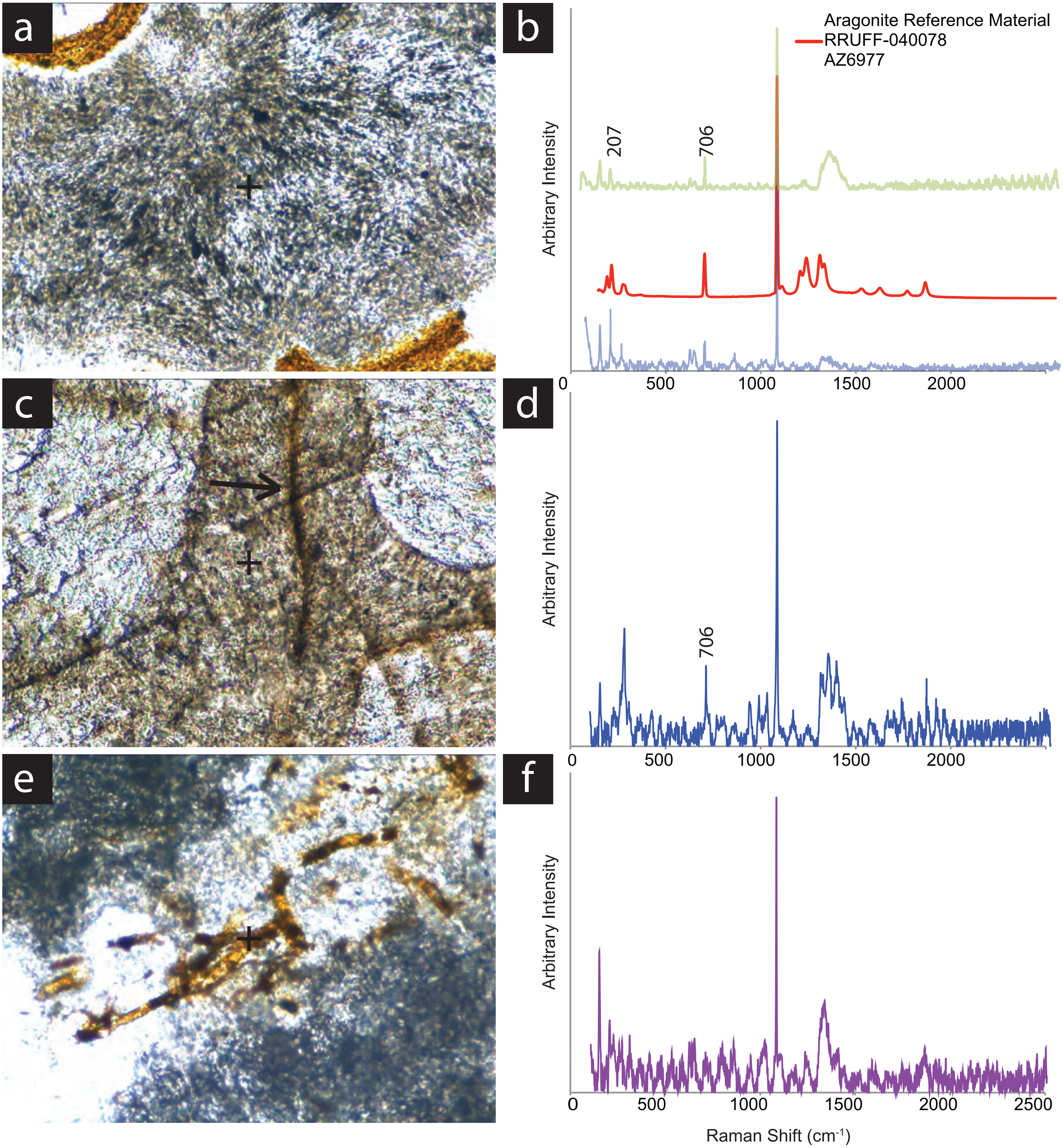

Figure S15- Analysis of coral fossil composition; thin section photomicrographs on the left (field of view = 340 μm) and Raman spectroscopy on the right. Raman spectra were generated at the location of the crosshairs on the photomicrographs and all spectra consistent with aragonite except F, which targets organics (see reference spectra in B). A) *Acropora papillare;* a Z-like Miocene coral. B) Raman spectra from *Acropora papillare* in A. C) *Gablonzeria* sp., a Z-like Triassic coral, note that organic material is disseminated throughtout the coral skeleton and concentrated in the centers of calcification on the septa (arrow). D) Raman spectra from *Gablonzeria* sp. in C. E) *Oulophyllia* sp. a Z-like Miocene coral with organic rich microborings. F) Raman spectra from microboring in *Oulophyllia* sp. in E. The peak around 1090 cm^{-1} is the Raman shift associated with the carbonate anion, while peaks 207 and 706 cm^{-1} are planar bending characteristic of aragonite[28].

Table S1- Results of Nitrogen Isotope Analyses of the Coral Organic Matrix

Sample	Mean Organic Matrix δ¹⁵N (‰)	Method Used	Age
Diploria labyrinthoformis-TR1-a	4.17	Dialysis/ combustion	Modern
Diploria labyrinthoformis-TR1-b	3.90		
Diploria labyrinthoformis-TR1-c	4.15		
Diploria labyrinthoformis-TR2-a	3.98		
Diploria labyrinthoformis-TR2-b	3.80		
Diploria labyrinthoformis-TR2-c	3.97		
Favia fragum-TR1-a	4.45		
Favia fragum-TR1-b	4.62		
Favia fragum-TR1-c	4.59		
Favia fragum-TR2-a	3.98		
Favia fragum-TR2-b	3.68		
Favia fragum-TR2-c	4.08		
Desmopyllum dianthus	9.49		
Desmophyllum dianthus	10.30	Persulfate/ denitrifier	
Diploria strigosa-TR1-a	5.71	Dialysis/ combustion	Holocene
Diploria strigosa-TR1-b	5.94		
Diploria strigosa-TR1-c	5.49		
Diploria strigosa-TR1-d	5.85		
Diploria strigosa-TR2-a	6.22		
Diploria strigosa-TR2-b	6.07		
Diploria strigosa-TR2-c	6.03		
Diploria strigosa-TR2	4.42	Persulfate/ denitrifier	
Oulophyllia sp.	6.19		Late Miocene
Acropora papillare	3.86	Dialysis/ combustion	Early Miocene
Acropora papillare	3.06	Persulfate/ denitrifier	
Caryophylllia sp. (1)	11.30		
Caryophyllia sp. (2)	10.70		
Antiguastrea lucasiana-TR1-a	4.33	Dialysis/ combustion	Oligocene
Antiguastrea lucasiana-TR1-b	3.99		
Antiguastrea lucasiana-TR1-c	3.65		
Antiguastrea lucasiana-TR2-a	4.35		
Antiguastrea lucasiana-TR2-b	3.95		
Antiguastrea lucasiana-TR2-c	4.13		
Distichophyllia norica-a	5.28	Dialysis/ combustion	Triassic (Rhaethian)
Distichophyllia norica-b	5.81		
Distichophyllia norica-c	5.81		
Retiophyllia norica-a	4.18		
Retiophyllia norica-b	5.15		
Retiophyllia norica-c	5.00		

Sample	Mean Organic Matrix $\delta^{15}N$ (‰)	Method Used	Age
Gablonzeria sp.-TR1-a	2.77	Dialysis/ combustion	Triassic (Norian)
Gablonzeria sp.-TR1-b	3.05		
Gablonzeria sp.-TR1-c	2.68		
Gablonzeria sp.-TR1	3.02	Persulfate/ denitrifier	
Gablonzeria sp.-TR2-a	4.21	Dialysis/ combustion	
Gablonzeria sp.-TR2-b	4.14		
Gablonzeria sp.-TR2-c	3.79		
Distichomeandra sp. (1)-TR1-a	4.83		
Distichomeandra sp. (1)-TR1-b	3.98		
Distichomeandra sp. (1)-TR1-c	5.15		
Distichomeandra sp. (1)-TR2-a	4.03		
Distichomeandra sp. (1)-TR2-b	4.30		
Distichomeandra sp. (1)-TR2-c	3.76		
Distichomeandra sp. (1)-TR2	4.16	Persulfate/ denitrifier	
Distichomeandra sp. (2)-TR1-a	2.38	Dialysis/ combustion	
Distichomeandra sp. (2)-TR1-b	3.57		
Distichomeandra sp. (2)-TR1-c	1.96		
Distichomeandra sp. (2)-TR2-a	1.90		
Distichomeandra sp. (2)-TR2-b	4.32		
Distichomeandra sp. (2)-TR2-c	4.42		

*Replicates of the same sample are labeled “TR1” and “TR2” respectively. Samples of the same species analyzed separately are labeled “(1)” and “(2).” Duplicates are labeled "a", "b," and "c."
